# Flax fiber based semicarbazide biosorbent for removal of Cr(VI) and Alizarin Red S dye from wastewater

**DOI:** 10.1038/s41598-023-34523-y

**Published:** 2023-05-22

**Authors:** Magda A. Akl, Abdelrahman S. El-Zeny, Mohamed A. Hashem, El-Sayed R. H. El-Gharkawy, Aya G. Mostafa

**Affiliations:** Present Address: Department of Chemistry, Faculty of Science, Mansoura University, Mansoura, 35516 Egypt

**Keywords:** Analytical chemistry, Chemical engineering, Environmental chemistry, Green chemistry, Materials chemistry, Polymer chemistry, Chemical synthesis

## Abstract

In the present study, flax fiber based semicarbazide biosorbent was prepared in two successive steps. In the first step, flax fibers were oxidized using potassium periodate (KIO_4_) to yield diadehyde cellulose (DAC). Dialdehyde cellulose was, then, refluxed with semicarbazide.HCl to produce the semicarbazide functionalized dialdehyde cellulose (DAC@SC). The prepared DAC@SC biosorbent was characterized using Brunauer, Emmett and Teller (BET) and N_2_ adsorption isotherm, point of zero charge (pH_PZC_), elemental analysis (C:H:N), scanning electron microscopy (SEM), Fourier transform infrared spectroscopy (FTIR) and X-ray diffraction (XRD) analyses. The DAC@SC biosorbent was applied for the removal of the hexavalent chromium (Cr(VI)) ions and the alizarin red S (ARS) anionic dye (individually and in mixture). Experimental variables such as temperature, pH, and concentrations were optimized in detail. The monolayer adsorption capacities from the Langmuir isotherm model were 97.4 mg/g and 18.84 for Cr(VI) and ARS, respectively. The adsorption kinetics of DAC@SC indicated that the adsorption process fit PSO kinetic model. The obtained negative values of ΔG and ΔH indicated that the adsorption of Cr(VI) and ARS onto DAC@SC is a spontaneous and exothermic process. The DAC@SC biocomposite was successfully applied for the removal of Cr(VI) and ARS from synthetic effluents and real wastewater samples with a recovery (R, %) more than 90%. The prepared DAC@SC was regenerated using 0.1 M K_2_CO_3_ eluent. The plausible adsorption mechanism of Cr(VI) and ARS onto the surface of DAC@SC biocomposite was elucidated.

## Introduction

The availability of accepted quality of water is one of the major problems faced in the twenty-first century^1^. The quality of water resources is declining daily due to various anthropogenic activities, unplanned urbanization, and increasing industrialization^2^.

Recently, chromium pollution has drawn great attention due to its harmful effects on the environment, human health and agriculture. There are two forms of chromium, trivalent chromium (Cr(III)) and hexavalent chromium (Cr(VI)). The hexavalent form is more toxic and carcinogenic than the trivalent. Chromium compounds especially Cr(VI) is dangerous to human health as it harms the digestive system, skin, and respiratory system. Cr(VI) enters various industrial fields like metal finishing, electroplating, atomic power plants, and metallurgy. The discharge limits of chromium have been developed by some industrial countries to reduce environmental pollution. WHO organization has instituted the total chromium maximum to be 0.05 mg/L^3^.

Alizarin Red S (ARS) is an anionic, non-biodegradable and water-soluble sodium salt that belongs to anthraquinone dyes. The inappropriate discharge of ARS into water bodies might have a negative impact on marine life. The discharge of ARS to the environment is considered as a direct threat to the ecosystem^4,5^.

Various types of dyes and metal ions are the major pollutants encountered in wastewater effluents, which disturb the aquatic environment^6,7^. So, the removal of water pollutants is an important issue.

Many technologies have been emerging for treating and handling pollutant-laden wastewater. Some commonly used treatment technologies consist of biological treatments, membrane process, chemical, and electrochemical technology, reverse osmosis, ion exchange, electrodialysis, electrolysis, and adsorption techniques^8^.

However, the limitations of most of these methods include the generation of toxic sludge, high operational and maintenance costs, and the intricate technique involved in the treatment^9^. Comparatively, the adsorption method is considered a better treatment process in wastewater treatment technologies due to ease of operation, convenience, and simplicity of design^10,11^. But it has a limitation of sludge generation (spent adsorbents after use) as other removal processes^12^.

In recent years, many attempts have been made to find inexpensive alternative materials as biosorbents that are both effective and acceptable for industrial use from both a technological (simplicity) and economic point of view^13^.

A recent class of materials proposed for environmental applications such as the removal of pollutants from solutions is based on the use of plants such as hemp and flax.

These two plants are annual, high-yielding crops grown for their fibers and seeds^14–16^. Hemp and flax are interesting raw, eco-friendly, lignocellulosic materials because of their ease of production (rapid growth, no pesticides), low cost, renewable character, particular chemical composition of their fibers (mainly cellulose, hemicelluloses and lignin), particular structure (multicellular fibers of fibrilar structure), physical and mechanical properties as well as their versatility. They are usable in the form of powder, fragments, fibers and oils since the entire plant (seeds and plant stem) is recoverable^14^. Like other lignocellulosic fibers or plant fibers such as jute, ramie, sisal, kenaf and bamboo, hemp and flax contain a high content of cellulose and comprise three main constituents (cellulose, hemicelluloses, and lignin) and other minor components.

As the main constituent of the flax fiber structure, cellulose builds the microfibrils in the cell wall structure, while hydroxyl groups on the C2, C3, and C6 positions in cellulose monomer units are responsible for the formation of a supermolecular structure with amorphous and crystalline regions by creating intramolecular and intermolecular hydrogen bonds between the cellulose chains^17^. Hemicelluloses, which contain hydrophilic hydroxyl groups, are connected to the cellulose by hydrogen bonds and are deposited in the interfibrillar spaces of the primary and secondary walls. Lignin is a complex three-dimensional polyphenolic polymer with a high molecular weight and low reactivity, deposited in the middle lamellae, and the secondary wall^18^. As mentioned previously, these main structural components contain specific functional groups (hydroxyl, carbonyl, and methoxyl)^19^, which, combined with the specific structure of flax fibers, promote their characteristics, especially their sorption properties.

One of the important features for the use of flax fiber-derived adsorbent for removal of dyes and metal ions is that it can be used on a large scale because of its huge availability. Also, biomass-derived adsorbents have high affinity/binding capacity for various types of contaminants, and also have good regeneration capacities. In addition, their good surface chemistry such as pore distribution, specific surface area, and functional groups help for the removal of dyes in the wastewater.

Cellulose is a traditional low-cost efficient adsorbent which has high potential for heavy metal removal from wastewaters due to its abundance, chemical and mechanical stability, high adsorption capability and unique structural properties. Cellulose has several advantages as it is cheap, renewable, available, regenerable, eco-friendly and biodegradable. Also, cellulose has a high uptake capacity and low density. Modifications of cellulose are performed to introduce functional groups. Cellulose is easily modified because of the presence of three hydroxyl groups in each cellulose unit. Cellulose is modified by several techniques like oxidation, esterification, etherification and halogenation^20–23^.

Flax has been proposed for removal of Zn, Cu and, Pb^24,25^, Cadmium^26,27^, uranium^19^, and Cu(II)^28^. Pejić et al. recently reported the role of cellulosic and noncellulosic functional groups in the biosorption of Pb(II) ions by waste flax fibers^29^. Furthermore, flax fiber has been proposed for removal of basic yellow 37 dye^30^ and recently for the removal of methylene blue, crystal violet and brilliant green cationic dyes by Akl et al.^31^. Liu et al. utilized Nano-TiO_2_ self-assembled flax fiber for oil/ water separation^32^.

In recent years, ion-pair formation has been used to remove various organic and inorganic contaminants. The advantages of ion-pair formation include higher reactive surface area and faster and more complete reactions^33^. However, there are still some technical challenges associated with practical applications such as limitations imposed by the high reactivity and low stability. Inspired by these findings and taking into consideration the ion-pair formation, this study aimed at the synthesis of N-donor modified flax adsorbent for removal of Cr(VI) and ARS via ion pair formation between N-donor modified flax fiber and Cr(VI) or ARS. Flax fiber was oxidized with periodate preceding its condensation with semicarbazide hydrochloride for the synthesis of the cationic DAC@SC functionalized semicarbazide-modified flax fiber.

To the best of our knowledge, no studies have been reported in the literature concerning the synthesis and use of DAC@SC biocomposite for the removal of ARS and Cr(VI).

The present study was carried out with the following objectives:

i. Design and characterization of cationic dialdehyde cellulose from flax fiber (DAC@SC) using various instrumental performances as elemental analysis, SEM, TEM, FTIR, ^1^HNMR, XRD and TGA.
ii. Batch sorption experiments utilizing Cr(VI) and anionic dye (ARS) as pollutants.
iii. Studying the optimum parameters like pH, the initial dye concentration, adsorbent dose, temperature, isotherms, thermodynamics and oscillation time.
iv. Comparative evaluation of removal efficiency (R%) of Cr(VI) and ARS, feasibility, and reusability of DAC@SC with other adsorbents.
v. Elucidation of the mechanisms involved in the process of adsorption of Cr(VI) and ARS onto DAC@SC biocomposite.

## Experimental

### Materials

**Figure 1:**
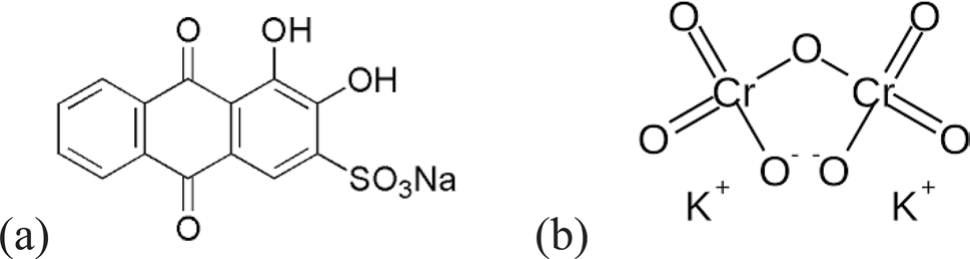
(**a**) Alizarin red S (ARS), (**b**) hexavalent chromium, Cr(VI).

Semicarbazide HCl (CH_5_N_3_O.HCl), potassium dichromate (K_2_Cr_2_O_7_), Alizarin red S (C_14_H_7_NaO_7_S), Fig. 1, tri-ethylamine (N(CH_2_CH_3_)_3_), hydroxyl amine hydrochloride (HONH_2_), absolute ethanol, sodium hydroxide (NaOH) and hydrogen chloride (HCl), were purchased from Sigma Aldrich. All the purchased chemicals were used without any pre-treatment. Flax fibers were supplied by Tanta flax & Oil Company, Egypt. Before use, flax fibers were washed by dist.H_2_O and were dried in an oven at 50 °C. Potassium periodate (KIO_4_) 4% solution was prepared by dissolving 4 g of KIO_4_ in 100 ml dist.H_2_O. Semicarbazide hydrochloride 2% solution was prepared by adding 2 g of semicarbazide.HCl to 100 mL absolute ethanol. Then, the mixture was heated until the semicarbazide hydrochloride is dissolved in ethanol.

### Instrumentation

The specific surface area of the DAC@SC biocomposite was obtained using the Brunauer Emmet Teller (BET) analysis (Size Analyzer (QUANTACHROME–NOVA 2000 Series).

The point of zero charge (pH_PZC_) is the pH value at which the biosorbent surface has a net zero charge. In this study, the pH_PZC_ was determined using the solid addition method^34^. In a series of 100 mL jacketed glasses, 50 mL of KNO_3_ solutions of known concentration were transferred. The solutions of different initial pH (pH_i_) between 2 and 12 were prepared by adding either 0.1 M HCl or 0.1 M NaOH. One gram of biosorbent was then added to each solution with stirring for 48 h. The final pH (pH_f_) was measured and the difference between the initial and final pH values (ΔpH = pH_i_− pH_f_) was plotted against pHi. The pHpzc value is the point where the curve ΔpH vs pHi crosses the line ΔpH = 0.

Estimation of the CNH composition for the DAC@SC biocomposite was achieved by a Costech (ECS-4010) elemental analyzer (Costech, Italy).

The surface morphologies of native, oxidized and modified flax fiber (DAC@SC) biocomposite were inspected by a scanning electron microscope (A JSM-6510LV).

The particle size of DAC@SC was measured using Transmittance electron microscopy, (TEM Talos f200i thermo scientific).

Fourier transform infrared (FTIR) spectra of the native flax fibers, DAC, DAC@SC biocomposite and the DAC@SC biocomposite having adsorbed the Cr(VI) and ARS were obtained by a Perkin–Elmer, Spectrum RX I using KBr pellets at wavenumber range from 4000 to 450 cm^−1^.

^1^HNMR spectra of the prepared DAC and DAC@SC cellulosic materials were measured in DMSO and trifluroacetic acid (TFA) in the NM R Lap-National Research Center, Dokky, Egypt using a Joel 500 MHZ Japan.

X-ray diffraction (XRD) patterns of the DAC@SC biocomposite were obtained by a PAN analytical X’Pert PRO diffractometer over the 2-theta (2θ) range from 10° to 40°.

The thermal stability of oxidized flax fiber and DAC@SC biocompositewere examined by thermogravimetric analysis (Berkin Elmer TGA 4000) at a heating rate of 15 °C/min from 30 to 800 °C.

A Shimadzu UV-2550 double-beam UV–Visible spectrophotometer with a 1 cm quartz cell was used for measurements of Cr(VI) and ARS at *λ*_max_ = 447 nm and 436 nm, respectively.

A pH meter (Hi 931401, HANNA, Portugal) was used to measure the pH of sample solutions.

### Preparations

#### Preparation of dialdehyde cellulose (DAC)

One gram of the native flax fiber was oxidized using 100 mL of 4% potassium periodate. The previous mixture was shaken for 1 h in complete darkness to form dialdehyde cellulose (DAC). The obtained DAC was washed several times with dist.H_2_O and was dried in an oven at 50 °C^31^.

##### Determination of aldehyde content

**Figure 2:**
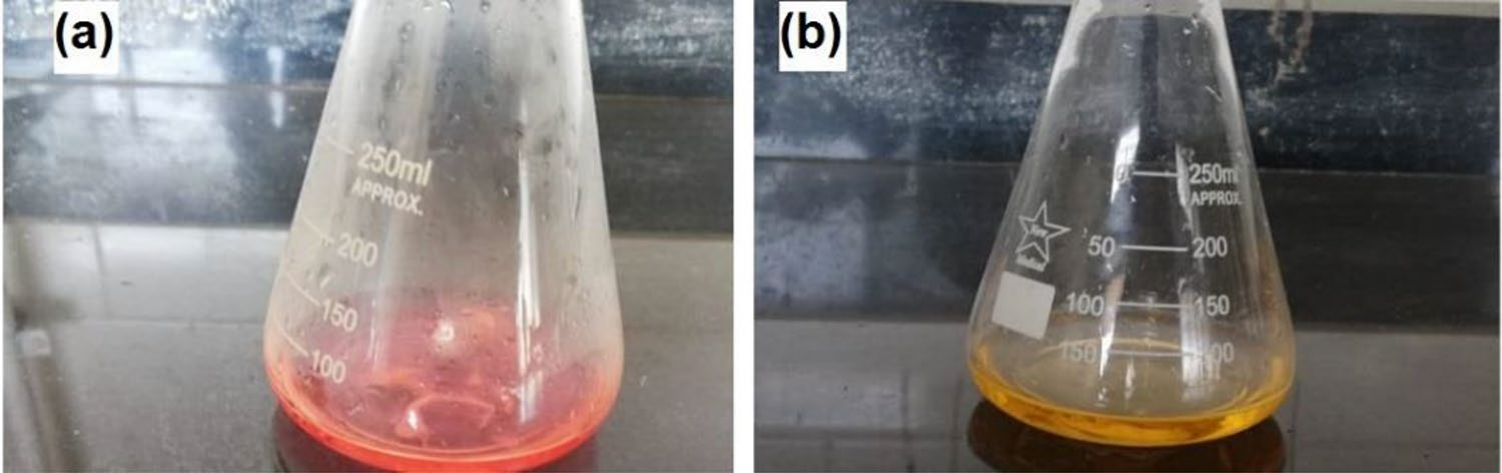
(**a**) Filtrate of 0.25 M hydroxylamine after stirring for 2.5 h with DAC; (**b**) after titration with 0.1 M NaOH.

0.25 M hydroxylamine HCl (1 L) was prepared as following: 17.55 g of hydroxylamine (99%) and 3 mL of 0.1% methyl orange indicator were added to 150 mL of dist.H_2_O. Then, the solution was completed to 1000 mL and its pH was adjusted to 4.0 by using 0.1 M NaOH^35^. 0.1 g of DAC was added to 25 mL of 0.25 M hydroxylamine HCl in a100 mL aluminum covered conical flask, Fig. 2, and was stirred at 25 °C for 150 min. Then, DAC was filtered and dried in an oven for 60 min at 100 °C. The filtrate was back titrated by using 0.1 M NaOH to pH 4 and the end point was achieved when the color is converted from red to yellow. The control experiment was performed by replacing the DAC with native flax fibers. The aldehyde content (A%) was calculated according to Eq. (1)^36^.

1 $${\text{AC}}\%=\frac{\text{NaOH Concentration }({\text{V}}_{\text{sample}}-{\text{V}}_{\text{control}})}{\text{m}/\text{Mwt}}X100$$

where AC% is the aldehyde content percentage, V_sample_ is the volume of NaOH in case of oxidized cellulose, V_control_ is the volume of NaOH in case of native flax fiber, m, and Mwt are sample weight and cellulose molecular weight, respectively.

#### Preparation of semicarbazide modified dialdehyde cellulose (DAC@SC) sorbent

**Figure 3:**
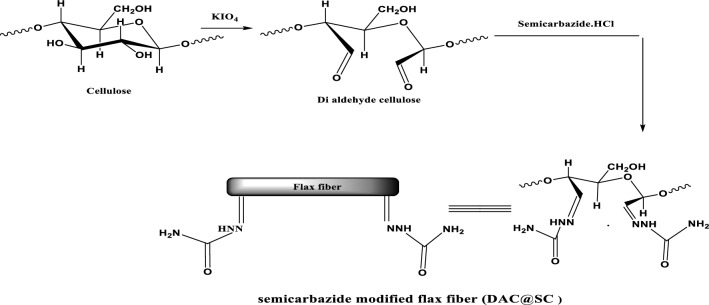
Synthesis of DAC@SC biocomposite.

One gram of oxidized flax fiber (DAC) was refluxed with 100 mL of 2% alcoholic semicarbazide.HCl and 0.5 mL tri-ethylamine for 4 h at 70 °C. Finally, the obtained modified DAC@SC biocomposite was washed several times with dist.H_2_O. The DAC@SC biocomposite was dried in the oven at 50 °C^37^. The synthesis of DAC@SC adsorbent is represented in Fig. 3.

### Biosorption and regeneration

The adsorption of Cr(VI) and ARS was carried out by batch experiments in triplicate samples in 125 mL stoppered bottles that contained 50 mL of adsorbate solutions and adsorbent dose. Then, the stoppered bottles were shaken at 150 rpm on the thermostated shaker at room temperature (25 °C). Sodium hydroxide and hydrochloric acid were used to adjust the pH of solution. The liquid supernatants that contain the remaining un-adsorbed Cr(VI) and ARS were measured. Various parameters were studied such as contact time ((15–140 min) for Cr(VI) and (30–180 min) for ARS), temperature (25–45 °C), adsorbent dose ((0.025–0.2 g) for Cr(VI) and (0.025–0.4 g) for ARS), pH (2–8), initial concentration of adsorbate ((50-350 mg/L) for Cr(VI) and (25-400 mg/L) for ARS) and ionic strength (0.0–0.2 mol/L) of sodium chloride.

The removal efficiency (R, %) and the amount of the adsorbed adsorbate at equilibrium q_e_ (mg/g) were determined using Eqs. (2) and (3), respectively.

2 $${\text{R}}\%=\frac{{\text{C}}_{\text{i}}-{\text{C}}_{\text{f}}}{{\text{C}}_{\text{i}}}\times 100$$

3 $${\text{q}}_{\text{e}}=\frac{\left({\text{C}}_{\text{i}}-{\text{C}}_{\text{f}}\right)\times \text{v}}{m}$$

where, C_i_ (mg/L) is the initial concentration of adsorbate, C_f_ (mg/L) is the equilibrium adsorbate concentration, m (wt, g) is the adsorbent dose, and V (L) is the adsorbate solution volume.

Regeneration of DAC@SC biocomposite was carried out by adsorption–desorption experiments. Cr(VI) adsorption experiments were carried out by using 0.1 g of DAC@SC and 50 mL (200 mg/L) of Cr(VI) solution at pH 2 for 100 min. ARS adsorption experiments were achieved by using 0.15 g of DAC@SC and 50 mL (100 mg/L) of ARS solution at pH 2 for 120 min. For desorption studies, 50 ml of eluent 0.1 M k_2_CO_3_ and 0.1 g of DAC@SC-Cr(VI) or 0.15 g of DAC@SC-ARS were shaken for 1 h. Finally, the regenerated adsorbents were used for another five repeated adsorption–desorption cycles.

### Preparation of real samples

#### Wastewater sample preparation

The digestion of wastewater samples was elaborated by using 0.5 g of potassium per sulphate, K_2_S_2_O_8_, and 5 mL of 98% (w/w) H_2_SO_4_ were added to 1000 mL of water sample and heated for 2 h at 95 °C to digest all organic matter which may be present. After cooling to room temperature, 0.1 g of DAC@SC modified fiber was added to the sample and the pH value was adjusted to 2 with continuous stirring for 180 min and filtered. Another 0.1 g of DAC@SC modified flax fiber was added to the filtrate to ensure the complete separation of analytes. The remains of Cr(VI) or ARS were determined by using Unicam UV 2100 UV/Visible spectrometer at appropriate wavelengths.

## Results and discussion

### Materials design

#### Synthesis of dialdehyde cellulose (DAC)

Potassium periodate is known as selective oxidizing agent which oxidizes two hydroxyl groups on two neighboring carbon atoms C_2_–C_3_ bond of the glucopyranoside ring that will be cleaved and converted into two dialdehyde groups. The oxidation degree which represents the percentage of monosaccharide units that reacted with periodate is calculated by aldehyde content determination^38^. The Aldehyde content of the prepared oxidized fiber is 35.71% as it is presented in Table 1.

**Table 1:** Volumetric titration of dialdehyde-cellulose (DAC) for determination of average aldehyde content percentage AC%:

V_control_ (mL)	V_sample_ (mL)	C_NaOH_ (M)	m (g)	AC %	Average AC%
0.5	2.7	0.1	0.1	35.2	35.71
0.5	2.7	0.1	0.1	35.2	
0.5	2.8	0.1	0.1	36.8	

#### Physicochemical properties of native flax fiber and DAC@SC biosorbent

Brunauer–Emmett–Teller (BET) surface area analysis was applied to evaluate the specific surface properties of the samples. The calculations of BET specific surface area showed that native flax fibers have a higher surface area (44.2151 m^2^/g) than that of the DAC@SC sorbent (10.3251 m^2^/g). The decrease of the specific surface area after chemical modification may be due to the covering of flax fiber pores by anchoring of semicarbazide moieties which reduced the adsorption of N_2_ molecules used in the surface area measurement process. The relatively low surface area of the functionalized fibers indicated that the adsorption process Occurs mainly through the coordination of the semicarbazide groups with the Cr(VI) ions and ARS.

The solubility of DAC@SC was investigated using different solvents such as ethanol 99.9%, HCl (0.1 M, 0.25 M, and 1 M) and NaOH (0.1 M, 0.25 M, and 1 M). It was evaluated that DAC@SC is not soluble in any used solvents.

#### Optical images

**Figure 4:**
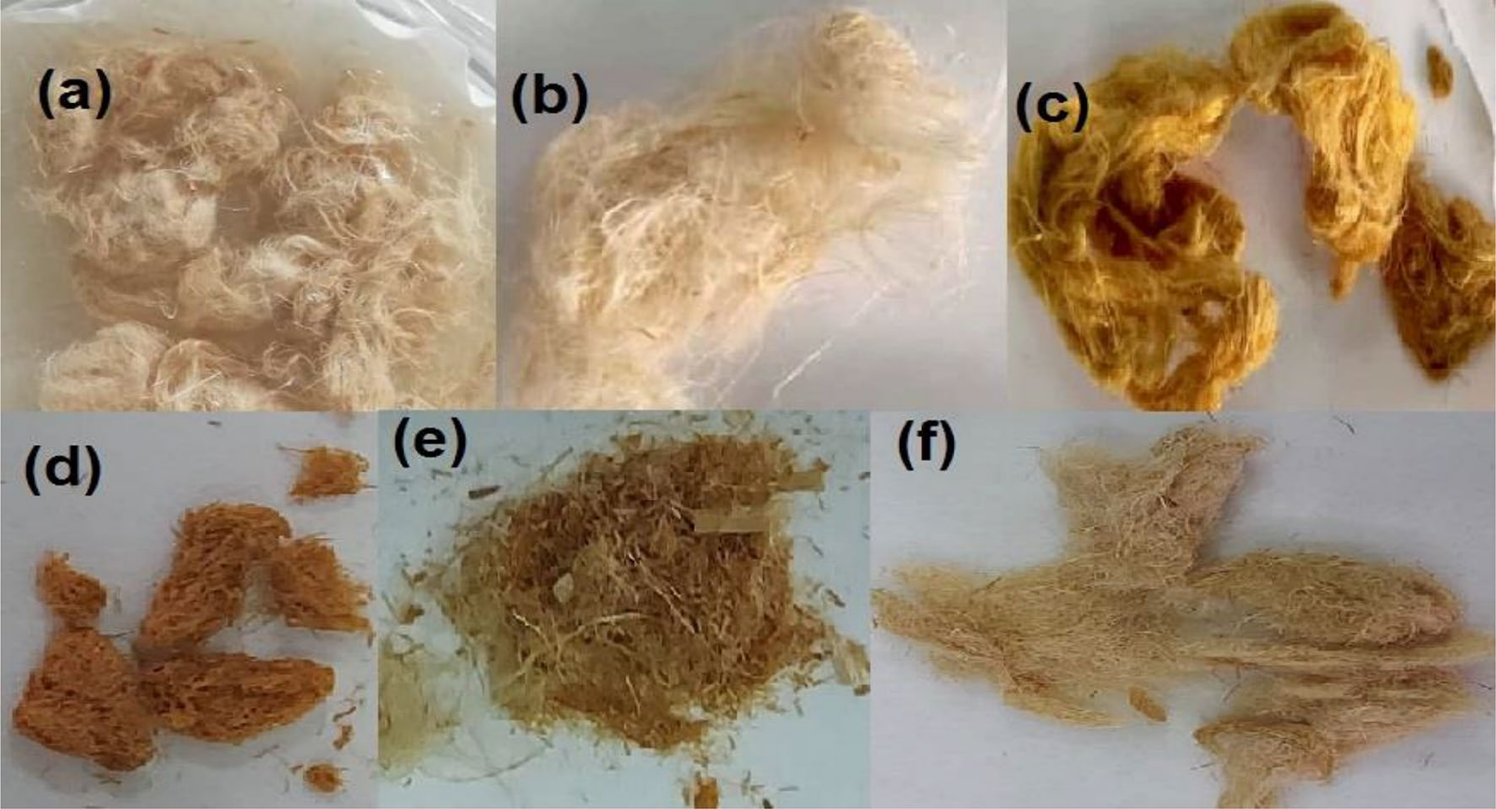
Optical images of (**a**) Native flax fibers, (**b**) oxidized flax fibers (DAC), (**c**) Semicarbazide modified flax fibers (DAC@SC), (**d**) DAC@SC-ARS, (**e**) DAC@SC-Cr(VI), (**f**) regenerated DAC@SC.

The optical images of native flax fiber, oxidized flax fiber (DAC), modified flax fiber (DAC@SC), the metal-loaded DAC@SC-Cr(VI), the anionic dye-loaded DAC@SC-ARS and the regenerated DAC@SC are shown in Fig. 4a–f, respectively. The images showed obvious color changes for flax fiber before (pale yellow) and after modification with semicarbazide (golden yellow).

### Characterization

#### Elemental analysis

The obtained results of elemental analysis for both native flax and DAC@SC materials are present in Table 2. The increase in nitrogen percent from 0% in the case of native flax to 11.33% in the case of DAC@SC biosorbent is an indication of DAC@SC biosorbent formation. The inserted semicarbazide moieties were calculated to be approximately 1.41 mmol/g.

**Table 2:** Elemental analysis of native flax fibers and DAC@SC biosorbent.

Fiber	C (%)	H (%)	N (%)
Native flax fiber	45	6.81	0
DAC@SC biosorbent	34.36	5.08	11.33

#### Morphology

**Figure 5:**
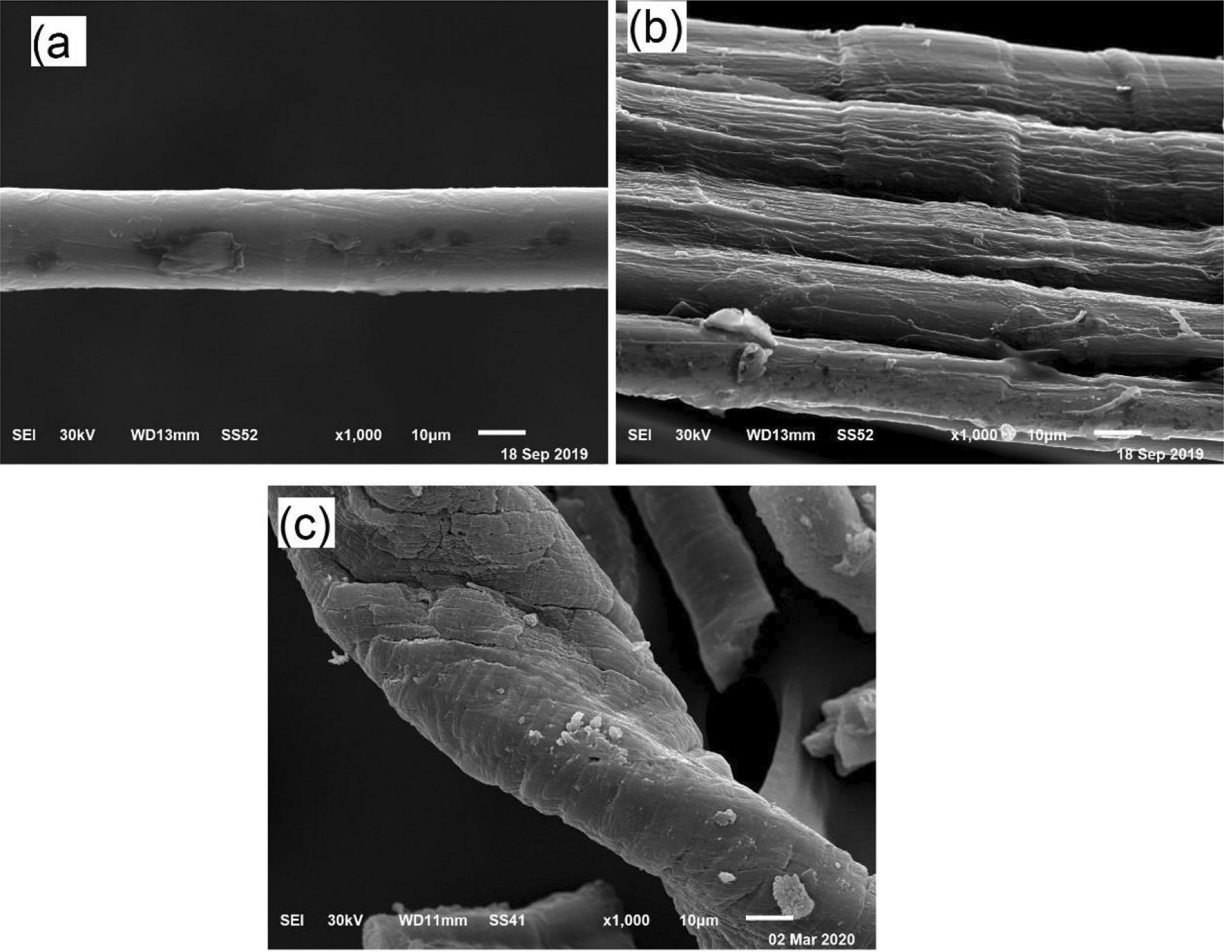
SEM of (**a**) native flax fiber, (**b**) oxidized flax fiber, and (**c**) DAC@SC biocomposite.

The surface morphology of native (Fig. 5a), oxidized (Fig. 5b) and DAC@SC modified flax (Fig. 5c) fibers has been detected by the scanning electron microscope. The presence of narrow strips on the oxidized fiber surface is detected as present in Fig. 5b and this may be returned to the periodate action during the oxidation process^39^. In Fig. 5c, the surface becomes rougher than the native and the oxidized which may be due to the chemical reaction between the semicarbazide.HCl and the oxidized flax fiber to form DAC@SC biocomposite.

#### Transmission electron microscopy (TEM)

**Figure 6:**
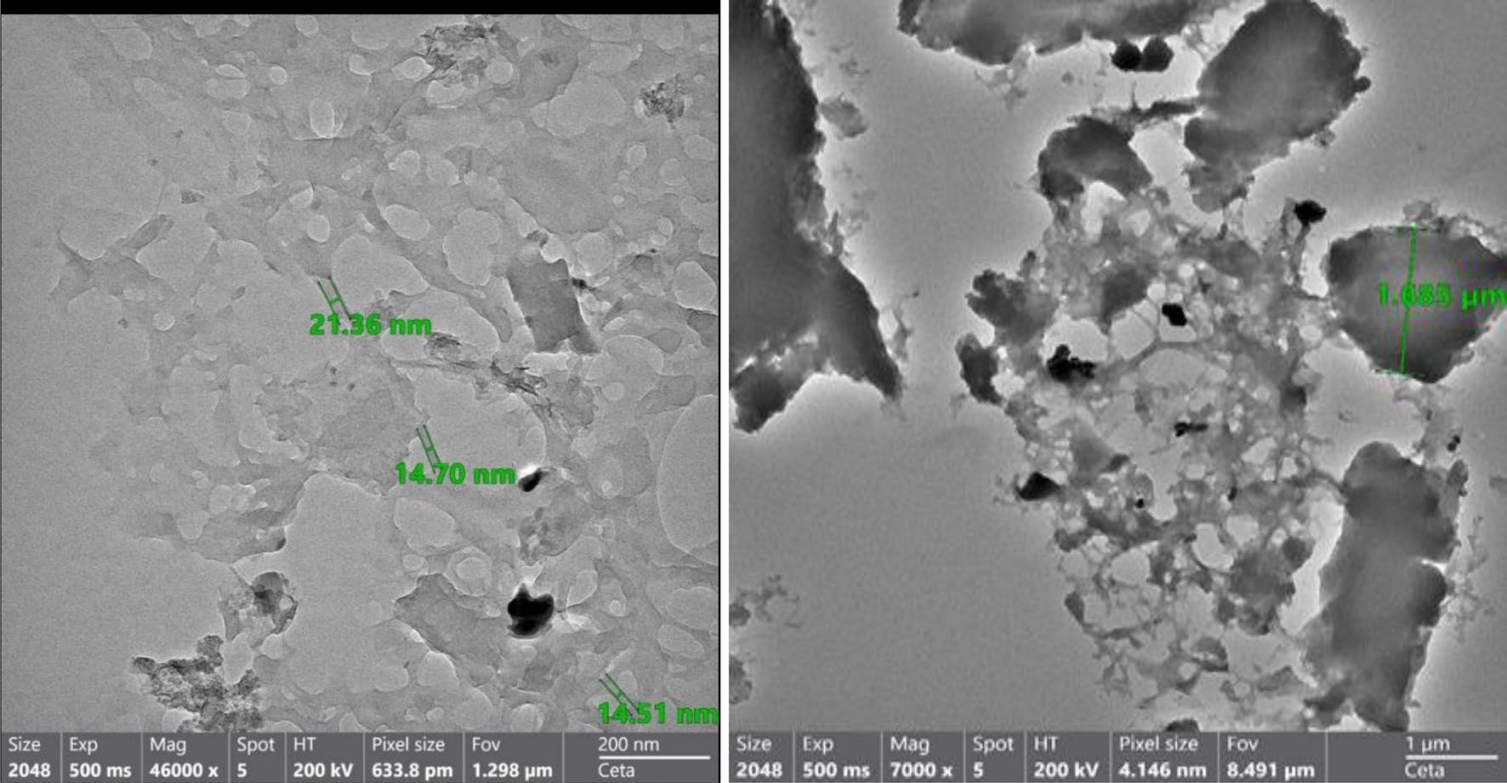
Transmission electron micrograph (TEM) of a cross section of DAC@SC composite.

Transmission electron microscopy (TEM) was used for investigating the internal shape of DAC@SC biocomposite. Figure 6 shows the formation of particles of size ranging between 14.51 nm and 1.685 µm. in addition to nondisintegrated fibrils that were also observed.

#### FTIR spectra

**Figure 7:**
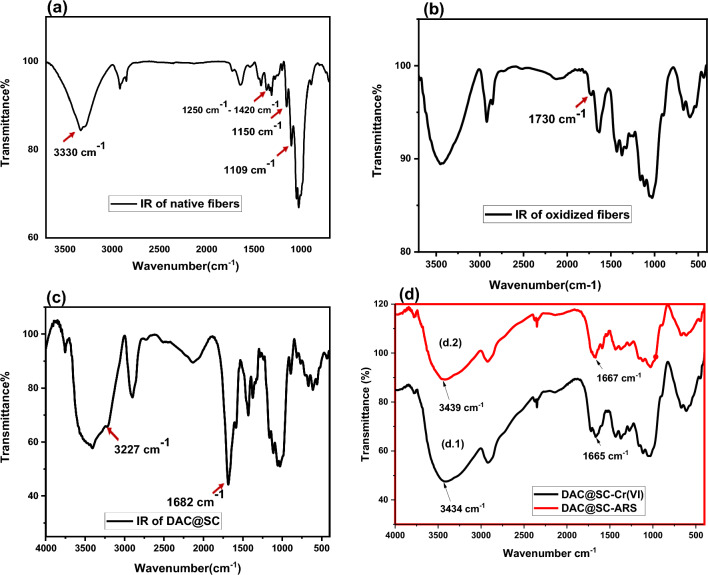
IR spectra of (**a**) native flax fiber, (**b**) oxidized flax fiber, (c) DAC@SC, (**d.1**) DAC@SC-Cr(VI), and (**d.2**) DAC@SC-ARS.

FTIR analysis was used to reveal one of the distinguishing characteristics of DAC@SC biocomposite, which is knowing the type of active functional groups present on DAC@SC; thus estimating the ability of DAC@SC biocomposite to adsorb the anionic pollutants. The progressive steps for the synthesis of the DAC@SC as chelating fibers were recognized by utilizing FT-IR spectra and the obtained results are displayed in Fig. 7.

Figure 7a,b show the IR spectra of both native and oxidized strands flax fibers. The IR spectra of native strands flax fiber (Fig. 7a) gives fingerprint peak of C–O stretching vibrations at around 1070–1150 cm^−1^, peaks of O–H bending vibrations at around 1250–1420 cm^−1^ and peaks of O–H stretching vibrations at around 3500–3200 cm^−1^.

The IR spectrum of oxidized flax fiber in Fig. 7b gives a new peak at around 1730 cm^−1^ which may be ascribed to aldehyde group stretching vibrations^40^.

After the successive reaction with semicarbazide, the spectrum of the prepared DAC@SC (Fig. 7c) shows a new peak at nearly 1680 cm^−1^ which can be ascribed to the azomethane group of the Schiff base which is formed between the dialdehyde groups of the oxidized flax fiber and the amino group of the semicarbazide and peak at nearly 3227 cm^−1^ which can be ascribed to the NH group of SC and broadening at 3404 cm^−1^ related to stretching vibration of the hydroxyl group and H bond of cellulose.

Figure 7d shows the IR spectra of DAC@SC-Cr(VI) and DAC@SC-ARS after loading . Slight shifts related to stretching vibration of the hydroxyl group can be observed from 3404 to 3434 cm^−1^ for DAC@SC-Cr(VI) and 3439 cm^−1^ for DAC@SC-ARS and the peak became broader. Also, the peak of the azomethane is shifted for from 1682 to 1665 cm^−1^ for DAC@SC-Cr(VI) and 1667 cm^−1^ for DAC@SC-ARS^36,39^.

#### ^1^H NMR

The most frequent analytical tools to study the structure of cellulose are solid and liquid phase nuclear magnetic resonance (NMR) while X-ray diffraction is appropriate for the characterization of the crystal forms^41^. Using solid phase ^13^C NMR also the different polymorphs of cellulose could be determined^42^, but it is less widely accessible and needs longer experiments than liquid phase NMR.

**Figure 8:**
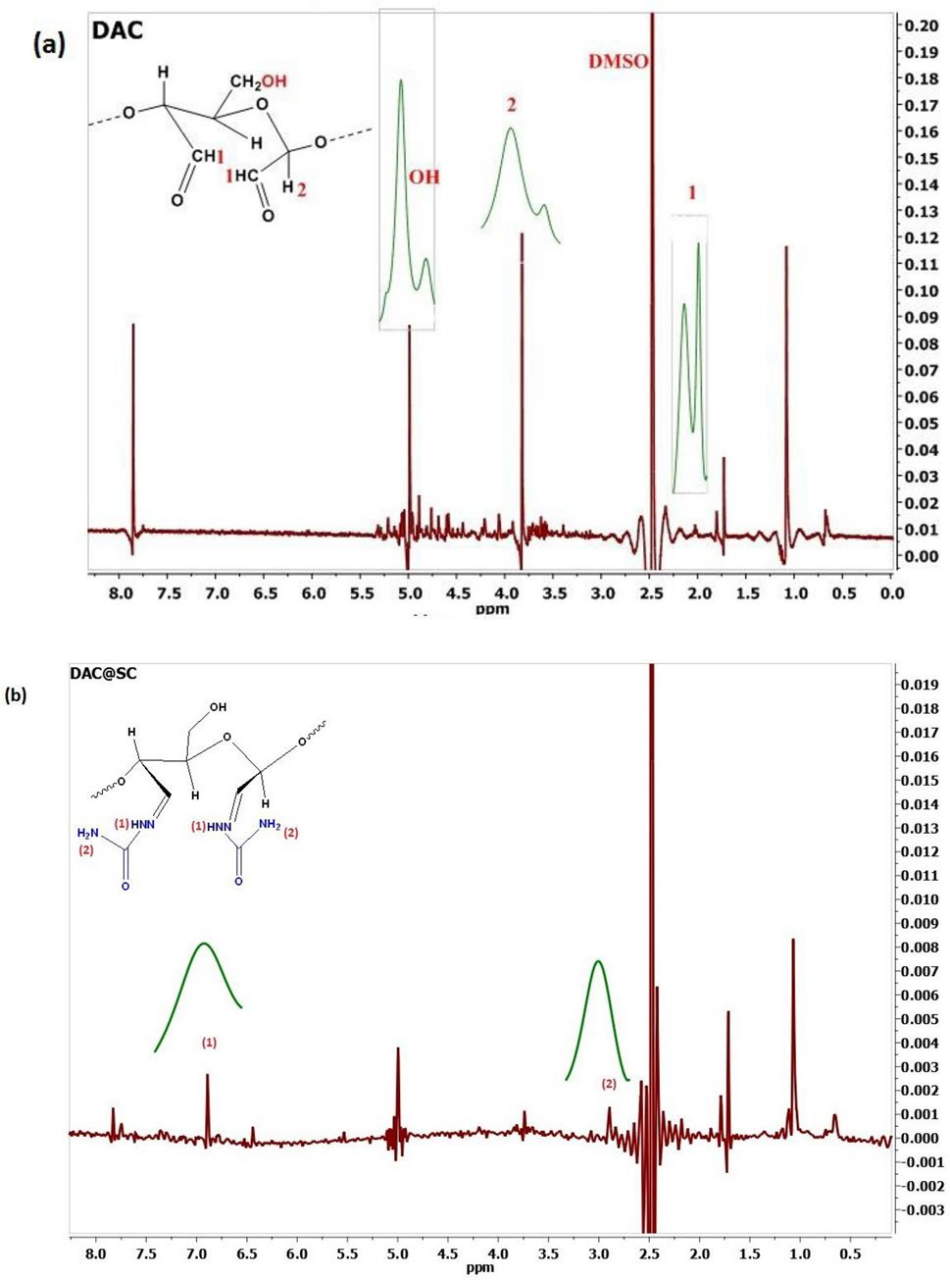
^1^H NMR of (**a**) DAC and (**b**) DAC@SC biocomposite.

The modified cellulose materials (DAC and DAC@SC) are insoluble in all molecular solvents^42^. Herein, we present the use of a DMSO/Trifluoroacetic acid mixture in the ^1^H NMR characterization of the DAC and DAC@SC biocomposite. ^1^H NMR confirmed the structure of the DAC and DAC@SC as present in Fig. 8. As an example, the ^1^H NMR of DAC present in Fig. 8a, showed a peak at 2.08 ppm is related to the proton that is present on C_2_ or C_3_. Broad peaks that appeared at 3.82 ppm and 4.98 ppm are related to ^1^H of C_1_ and OH, respectively. Figure 8b represents ^1^H NMR of DAC@SC biocomposite that showed new prominent peaks, broad signals around 2.91 ppm and 6.89 ppm that are attributed to NH and NH_2_ of semicarbazide, respectively.

#### X-ray diffraction

**Figure 9:**
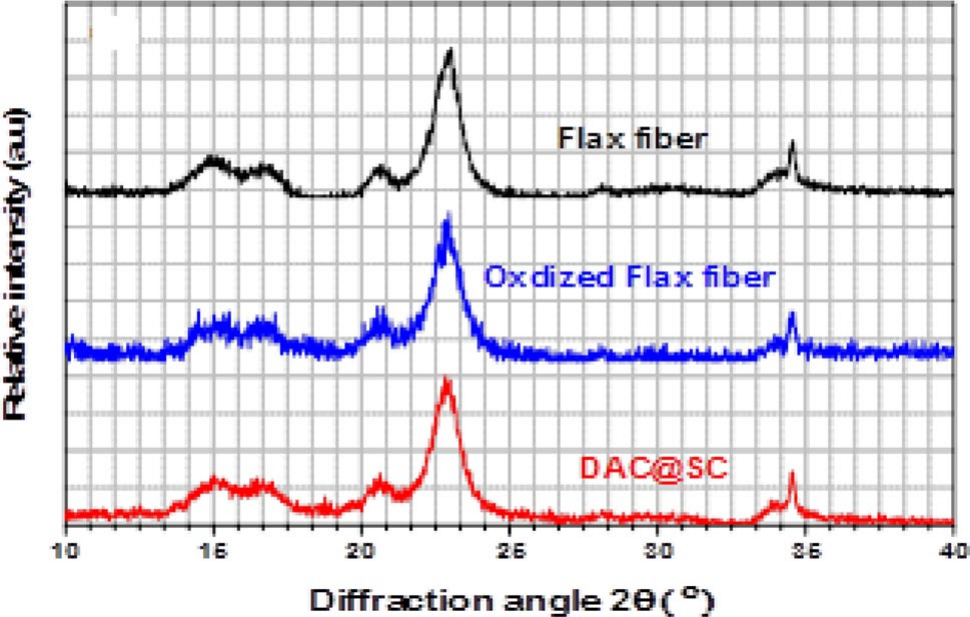
XRD patterns of flax, oxidized flax and DAC@SC samples.

XRD pattern of native, oxidized and modified flax fiber are shown in Fig. 9. The XRD patterns illustrate the main peak of crystalline flax at 22.835° which is related to the (200) reflection. Moreover, the less intense peaks arising at about 15.178°, 16.756°, and 34.475° are the characteristic of (11̅0), (110) and (004) reflections, respectively^43–45^. The peak around 2θ≈21 could be decrease in crystallinity due to amorphous part of the prepared modified flax fibre^43^. The decrease of crystallinity is due to the ring-opened formation of the glucopyranose units and destruction of their backbone. The crystallinity indices (CrI) of the samples were calculated according to the Segal method^46^.

4 $${\text{CrI}}\% =\frac{{{{I}}_{200}} \, - \, {{{I}}_{\text{am}}} \, }{{{{I}}_{200}}}\times \, 100$$

where *I*_200_ is the intensity of the crystal peak at the maximum 2*θ* = 22.835° and *I*_*am*_ is the intensity at the minimum at 2*θ* = 15.185°. The crystallinity indexes of native, oxidized and modified flax fibers are 73.19, 71.77, and 71.24%, respectively. These results reveal that the ordered structure of crystalline flax fiber is not significantly altered after the modification process of flax fibers.

#### Thermogravimetric analysis (TGA)

**Figure 10:**
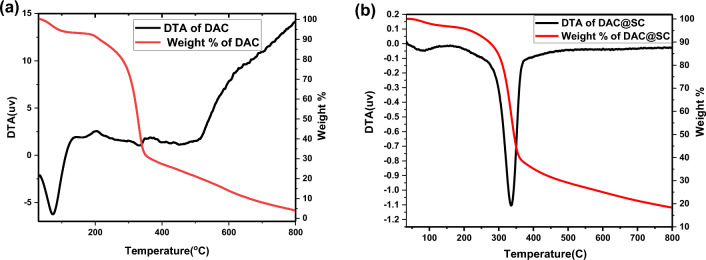
Thermal analysis of (**a**) DAC and (**b**) DAC@SC composite.

The TGA curves of oxidized cellulose and DAC@SC biocomposite samples were shown in Fig. 10. For oxidized flax fiber, Fig. 10a, the sample shows an initial weight loss ranging from 80 to 250 °C that would be related to the evaporation of the adsorbed water molecules. The second weight loss gradually starts at 250 °C which leads to a sharp fall at 330–400 °C. This would be corresponding to the degradation of the holocellulose content of flax fibers degradation and another degradation that appeared at 480 °C was observed. Oxidation of cellulose leads to a variation in the structure, crystallinity, and degree of polymerization, which in turn affect its physical and chemical properties^47,48^.

While for DAC@SC, Fig. 10b, the first degradation step for DAC@SC biocomposite begins at 30 °C until 265 °C, which was due to the evaporation of the residual water present in cellulose components^49^. The second degradation step occurred from 265 to 371 °C, DAC@SC biocomposite displayed gradual thermal transitions, basically related to the cellulosic chain degradation^50^. The major decomposition happened 272–372 °C range which indicates the pyrolysis of cellulose^51^. At 800 °C, the ash content of DAC powder is 4.1% increased to 18.3% in the case of DAC@SC biocomposite with the loading of semicarbazide, which proves increasing the thermal stability of the oxidized fibers with the loading of semicarbazide.

### Adsorption studies

#### Point of zero charge (pH_PZC_)

The point of zero charge of the biosorbent is one way to understand the biosorption mechanisms. The surface charge of DAC@SC biocomposite was evaluated by measuring the pH at the point of zero charge (pH_PZC_). Generally, the adsorbent will exhibit better affinities for anions at pH < pH_PZC_ and vice versa. The pH_PZC_ value obtained for DAC@SC biocomposite was approximately 6.04. It was expected that the adsorption of Cr(VI) and ARS could be enhanced at the experimental pH (pH 2) due to electrostatic interaction between the main species of Cr(VI), Cr_2_O_7_^2−^, HCr_2_O_7_^−^, and SO_3_^−^ of ARS and nitrogen-containing functional groups such as –NH_3_^+^ on the surface of DAC@SC^52,53^. Similar results were reported by Ma et al., in which their research observed that the electrostatic attraction between Cr(VI) and protonated amino groups increased the Cr(VI) removal efficiencies under acidic conditions^54^. In addition, the composition of flax fibers comprises abundant oxygen-containing functional groups such as –OH. Because of the protonation effect, the surface of adsorbents will have positive charges below pH_PZC_, thus improving the attraction for anions^55^.

#### Effect of pH

**Figure 11:**
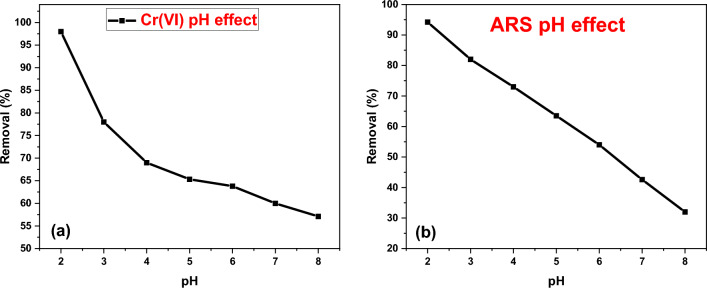
Effect of pH on adsorption of (**a**) Cr(VI) and (**b**) ARS (conditions: 0.1 g of DAC@SC in 50 mL of the 100 mg/L Cr(VI) solution. 0.15 g of DAC@SC in 50 mL of the 100 mg/L ARS aqueous solution at 25 °C for 2 h).

The initial pH of the mixture solution plays an important role in biosorption of analytes due to its impact on the active binding sites of the biosorbent and species distribution of the metal ions and dyes^56,57^. The adsorption of Cr(VI) and ARS are strongly pH dependent. So, the studying of pH parameter is very important. The pH parameter for Cr(VI) adsorption was studied by adding 0.1 g of DAC@SC to 50 mL of the 100 mg/L Cr(VI) aqueous solution at 25 °C for 2 h. ARS adsorption pH parameter was studied by adding 0.15 g of DAC@SC biocomposite to 50 mL of the 100 mg/L ARS aqueous solution at 25 °C for 2 h. In pH range (2–8), the Cr(VI) and ARS uptake were examined as a function of hydrogen ion concentration as present in Fig. 11a,b, respectively. It was observed that the maximum adsorption capacity was at pH 2. Experimental data confirms that the adsorption capacity for Cr(VI) and ARS are highly pH-dependent.

As the initial pH increased from 2 to 4, the removal % of Cr(VI) ions and ARS dye decreased very sharply and after that it decreased gradually throughout the process at around 20.0% and 15% for Cr(VI) and ARS, respectively. A similar trend was also observed with the removal of hexavalent chromium using other biosorbents including activated carbon developed from date palm seed, invasive biomass Sargassum muticum, chufa corm peels, and walnut hull^58,59^.

Hexavalent chromium ions exist in different forms in an aqueous solution such as HCrO_4_^−^, Cr_2_O_7_^2−^, CrO_4_^2−^and the stability of these forms depends mainly on the pH of the medium. It is well known that at very low pH values (pH < 4), the dominant anionic chromium species is the acid chromate ion (HCrO^4−^)^60^. In this pH range, the surface of biosorbent, which is highly positively charged due to protonation of functional groups of adsorbent, attracts strongly HCrO^4−^. However, as the pH increased, the predominant form HCrO_4_^−^ shifts to CrO_4_^2−^ and Cr_2_O_7_^2−^ ions and also the degree of protonation of the biosorbent surface reduces gradually. So, the lower adsorption capacity observed from pH 4.0 may be explained by the competition of these anionic ions with OH^−^ ions to be adsorbed on the surface of the adsorbent of which OH^−^ predominates^61^.

For the following adsorption experiments, pH 2.0 was selected as the optimum pH value for Cr(VI) solution. It was also observed that the decreasing of adsorption capacity with pH increasing from 2 to 8.

Because of the strong protonation, the adsorbent surface becomes positively charged at low pH. The Cr(VI) adsorption was enhanced due to the electrostatic force between negatively-charged HCrO^4−^ and Cr_2_O_7_^–2^ and SO_3_^−^ of ARS and the positively charged adsorbent surface. Thus, this, in turn, enhances the affinity of DAC@SC biocomposite towards attracting positively charged metal ion and dye molecules depending on pH control, causing the improvement of Cr(VI) and ARS adsorption as shown in the equations below (5) and (6), respectively.

5 $${\text{DAC}}@{\text{SC}}^{ + } + \, \left( {{\text{ARS}}} \right) \, - {\text{ SO}}_{{3}}^{ - } \leftrightarrow {\text{DAC}}@{\text{SC}}^{ + } \ldots {\text{SO}}_{{3}}^{ - } \left( {{\text{ARS}}} \right)$$

6 $${\text{DAC}}@{\text{SC}}^{ + } + \, \left( {{\text{Cr}}\left( {{\text{VI}}} \right)} \right) - {\text{O}}^{ - } \leftrightarrow {\text{DAC}}@{\text{SC}}^{ + } \ldots {\text{O}}^{ - } \left( {{\text{Cr}}\left( {{\text{VI}}} \right)} \right)$$

#### Effect of sorbent dose

**Figure 12:**
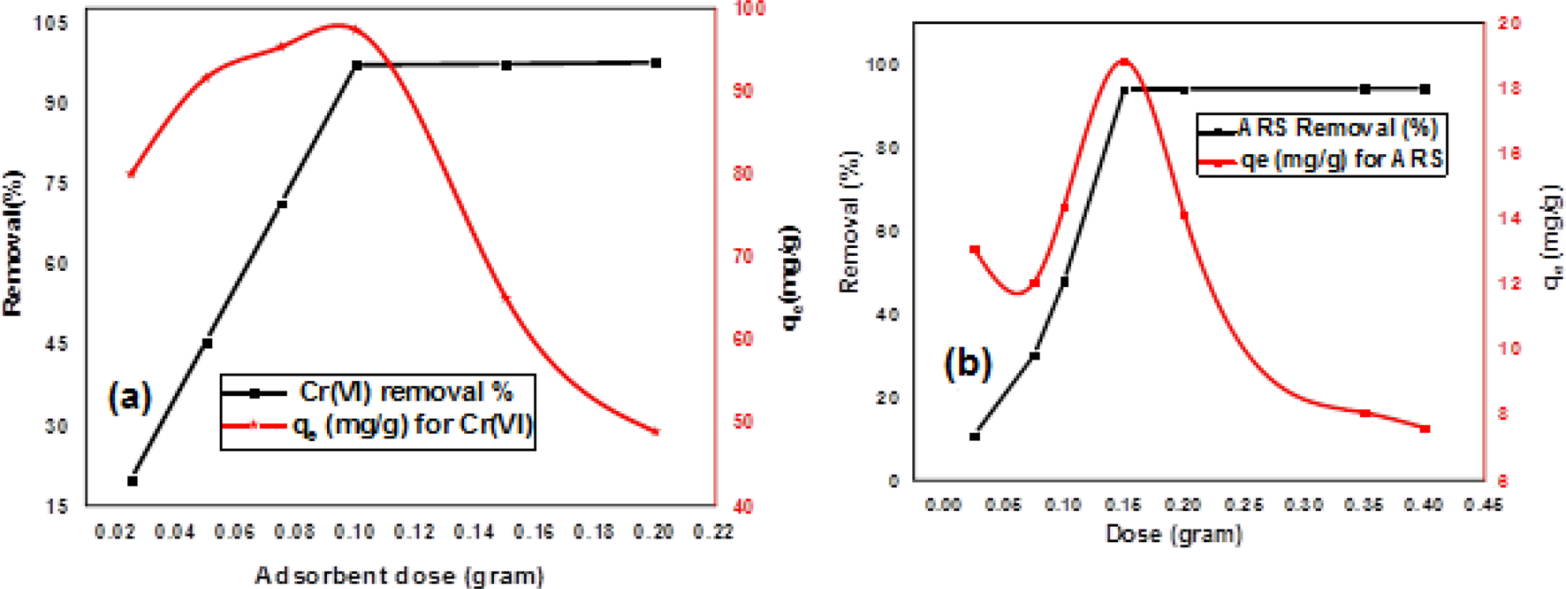
Effect of sorbent dose on adsorption of (**a**) Cr(VI) and (**b**) ARS(conditions: 50 mL aqueous solution of 200 mg/L for Cr(VI) solution and 100 mg/L for ARS solution at 25 °C for 2 h at pH 2).

Adsorbent dose is very important parameter in adsorption capacity determination. It was investigated by adding various weights of DAC@SC adsorbent in 50 mL aqueous solution of 200 mg/L for Cr(VI) solution and 100 mg/L for ARS solution at 25 °C for 2 h at pH 2. It was observed that the removal efficiency and adsorption capacity of Cr(VI) increased from 20 to 97.4%, and from 80 to 97.4 mg/g, respectively when the adsorbent dose from 0.025 to 0.1 g As the adsorbent dose was increased from 0.1 to 0.2 g, the removal efficiency showed few changes and adsorption capacity decreased from 97.4 to 48.925 mg/g as it is depicted in Fig. 12a. It is also observed that removal % of ARS increased from 10.9 to 94.13% and adsorption capacity increased from 13.08 to 18.83 mg/g with increasing the adsorbent dose from 0.025 to 0.15 g. With increasing the adsorbent dose from 0.15 to 0.4 g, the removal % showed slightly increasing and adsorption capacity decreased from 18.83 to 7.06 mg/g as it is depicted in Fig. 12b. This may be due to the increase in the specific surface area of the adsorbent and the presence of more available adsorption sites.

#### Effect of initial concentration of Cr(VI) and ARS

**Figure 13:**
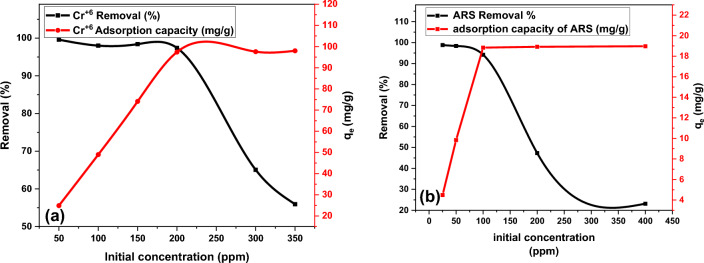
Effect of sorbent dose on adsorption of (**a**) Cr(VI) and (**b**) ARS (conditions: 0.1 g of DAC@SC for Cr(VI) and 0.15 g of DAC@SC for ARS were taken at pH 2 for 2 h in range 50–350 ppm for Cr(VI) and 25–400 ppm for ARS).

To study the effect of initial concentration on Cr(VI) and ARS adsorption capacity, a 50 mL solution of Cr(VI) and ARS at a fixed-dose of DAC@SC adsorbent 0.1 g for Cr(VI) and 0.15 g for ARS were taken at pH 2 for 2 h in range (50–350 ppm for Cr(VI) and 25–400 ppm for ARS). After that, initial concentrations were varied and the corresponding adsorption capacities and removal percentages were evaluated as shown in Fig. 13a,b. It was noticed that the adsorption capacity increased from 24.9 to 97.4 mg/g, and from 4.49 to 18.83 mg/g for Cr(VI) and ARS, respectively. With initial concentration increasing from 50 to 200 ppm for Cr(VI) and from 25 to 100 ppm for ARS. Moreover, with the increasing of initial concentration from 200 to 350 ppm for Cr(VI) and from 100 to 400 ppm for ARS, the adsorption capacities remained constant.

#### Biosorption isotherms

The adsorption isotherm of chromium (VI) ions and ARS onto DAC@SC biocomposite was studied by varying the initial concentration of metal from 30 to 300 mg/L, keeping other factors unchanged. The equilibrium biosorption was modeled using the Langmuir, Freundlich, and Dubinin–Radushkevich isotherm equations. The coefficient of determination, R^2^, was employed to ascertain the fit of these isotherms with experimental data.

The Langmuir isotherm is based on the assumption of monolayer coverage of adsorbate over a homogenous adsorbent surface with a finite number of adsorption sites.

The model equation is represented by the following linearized equation:

7 $$\frac{{{\text{C}}_{{\text{e}}} }}{{{\text{q}}_{{\text{e}}} }} = \frac{1}{{{\text{k}}_{{\text{L}}} {\text{q}}_{{\text{m}}} }} + \frac{{{\text{C}}_{{\text{e}}} }}{{{\text{q}}_{{\text{m}}} }}$$

The Freundlich isotherm assumes a heterogeneous surface of the adsorbent and linearized form of the model is as follows:

8 $$\text{ln}{q}_{e}={\text{ln}}{K}_{f}+\frac{1}{n}{\text{ln}}{C}_{e}$$

The D–R isotherm model assumes that biosorption is related to surface porosity and pore volume, and examines biosorption energetically. The mean free energy of biosorption (EDR) obtained from the d–R model determines whether the adsorption structure is chemical or physical. 8< EDR < 16 kJ mol^−1^ indicates that the adsorption has a chemical character. If EDR < 8 kJ mol^−1^, it determines that the adsorption is physical^13,62^.

The model equation is represented by the following linearized equation:

9 $${\text{lnq}}_{e}=\text{ ln}{\text{q}}_{m} -\text{ k}{\upvarepsilon }^{2}$$

where, Ce (ppm) is the initial concentration of the studied pollutant at equilibrium, q_e_ (mg/g) is the capacity of the adsorbent for pollutant concentration at equilibrium, q_m_ (mg/g) adsorption maximum amount, 1/n, K_L_, K_F_, and K are heterogeneity factor, Langmuir coefficient (L/mg), Freundlich constant (mg g^-1^), and the Dubinin–Radushkevich constant, respectively. While ε is the adsorption potential and is given by Eq. (10).

10 $$\upvarepsilon =\text{RTln}\left(1+\frac{1}{{\text{C}}_{\text{e}}}\right)$$

where R (8.314 J/mol K) is the gas constant, and T is the temperature in kelvin.

**Figure 14:**
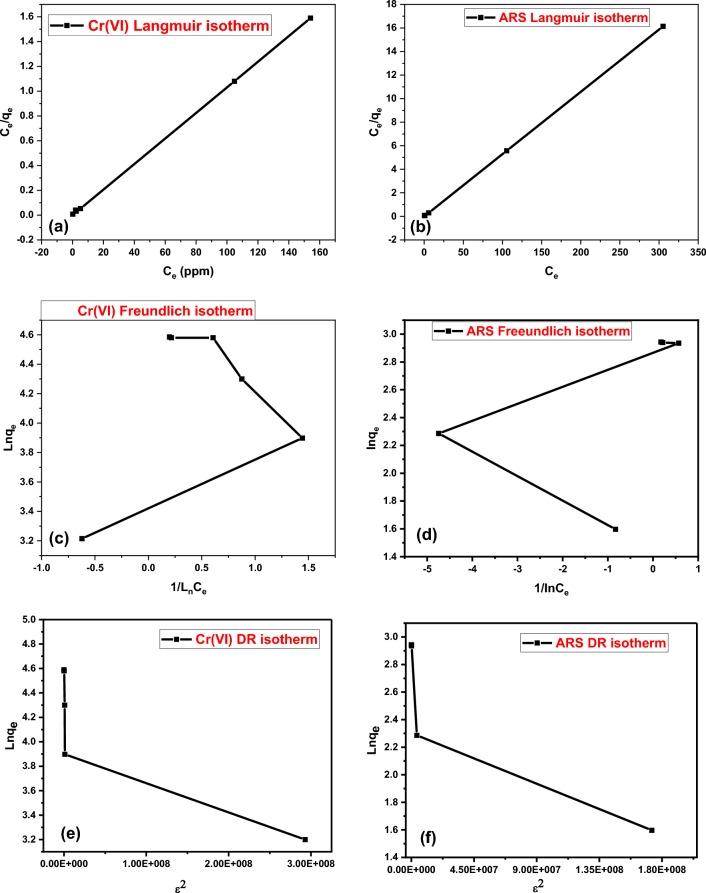
Biosorption isotherms by DAC@SC: (**a**) Langmuir isotherm model for Cr(VI), (**b**) Langmuir isotherm model for ARS, (**c**) Freundlich isotherm model for Cr(VI), (**d**) Freundlich isotherm model for ARS, (**e**) DR isotherm model for Cr(VI), and (**f**) D-R isotherm model for ARS.

The Langmuir, Freundlich and D-R isotherms determined for the biosorption of Cr(VI) and ARS to the DAC@SC biosorbent are shown in Fig. 14 and their derived parameters are given in Table 3. The Langmuir isotherm model (R^2^ = 0.999) seemed to describe better the adsorption process of Cr(VI) and ARS by the DAC@SC biocomposite than the Freundlich isotherm model (R^2^_(Cr(VI)_ = 0.1272, R^2^_(ARS)_ = 0.2027) This shows that the Cr(VI) and ARS molecules bind to the active sites on the biosorbent surface as a mono-layer. The maximum biosorbent capacity of Cr(VI) and ARS are 97.4mg/g, and 18.83 mg/g, respectively. The E_DR_ of biosorption from the D-R model was calculated as 11.083 and 8.512 kJ mol^−1^ for Cr(VI) and ARS, respectively. These values suggest that the bio-sorption process of Cr(VI) and ARS onto the DAC@SC biocomposite may be carried out by a mechanism being chemical in nature because the sorption energy lies within 8–16 kJ mol^−1^show that the Cr(VI) and ARS biosorption process on the biosorbent is chemisorption^66^.

**Table 3:** Biosorption isotherm parameters of Cr(VI) and ARS by DAC@SC.

Fiber	Langmuir isotherm constants			
	K_L_ (L/g)	q_m_ (mg/g)	R^2^	R_L_
DAC@SC-Cr(VI)	1.245	97.46	0.9999	0.0040
DAC@SC-ARS	2.83	18.939	0.999	0.00418

Fiber	Freundlich isotherm constants			
	K_F_	n	R^2^	
DAC@SC-Cr(VI)	1.314	0.2457	0.1272	
DAC@SC-ARS	14.2	8.162	0.2027	

Fiber	D–R isotherm constants			
	K	E (kJ/mole)	Qm (mg/g)	R^2^
DAC@SC-Cr(VI)	4.07 × 10^–9^	11.083	80.64	0.767
DAC@SC-ARS	6.9 × 10^–9^	8.512	16.11	0.79457

The essential characteristics of the Langmuir isotherm parameters can be used to predict the affinity between the sorbate and sorbent using separation factor or dimensionless equilibrium parameter, “R_L_”, expressed as in the following equation^63^.

11 $${R}_{l }= \frac{1}{1+{K}_{l}{C}_{\text{o}}}$$

In the present study, the R_L_ values obtained for all initial concentrations of metal ions lie between 0 and 1 (Table 3), indicating that biosorption of Cr(VI) ions and ARS by DAC@SC is a favorable process. This suggests the applicability of this biosorbent for Cr(VI) ion removal from aqueous solutions.

#### Effect of oscillation time and adsorption kinetics

**Figure 15:**
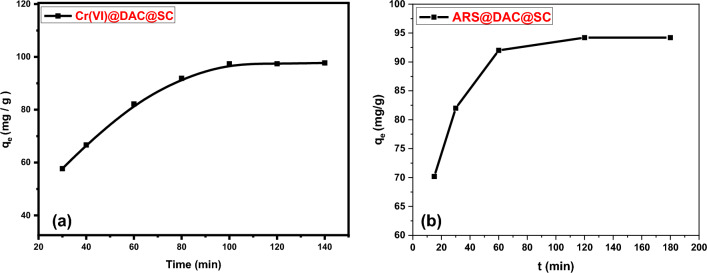
Effect of oscillation time on adsorption of (**a**) Cr(VI) (conditions: 0.1 g of DAC@SC, 50 mL of 200 mg/L Cr(VI), Temp.: 25 °C, time: 30–140 min). (**b**) ARS (conditions: 0.15 g DAC@SC, 50 mL of 100 mg/L of ARS, Temp.: 25 °C and time: 15–180 min).

Oscillation time is an important parameter for the investigation of sorption efficiency. The contact time parameter for Cr(VI) adsorption was studied at different times from 30 to 140 min by using 0.1 g of (DAC@SC) material as an adsorbent dose that were respectively added to a series of bottles that contain 50 mL of 200 mg/L Cr(VI) solutions at 25 °C as shown in Fig. 15a. Contact time parameter for ARS adsorption was studied at different time from 15 to 180 min by adding 0.15 g of (DAC@SC) adsorbent to a series of bottles that contain 50 mL of 100 mg/L of ARS solutions at 25 °C as shown in Fig. 15b. It is clear that the adsorption capacity of DAC@SC increased rapidly with the increase of contact time from 20 to 120 min and more than 90% of the equilibrium adsorption capacity for the two analytes occurred within 100 min. At 120 min, the adsorption capacity became constant and the adsorption reached equilibrium. As shown, the adsorption process was divided into three stages: (1) an initial stage with adsorption occurring instantly; (2) subsequently slow adsorption and (3) a final stage with adsorption reaching equilibrium and remaining constant. The first stage can be attributed to the rapid attachment of the Cr(VI) and ARS to the surface of DAC@SC by surface mass transfer. At this stage, more than 80% of adsorption was found in the two cases. The second stage was slower, possibly because many of the available external sites were already occupied and because of the slow diffusion of analyte molecules into the network of DAC@SC.

Parameters obtained by adsorption kinetic studies provide information about the determination of the adsorption rate, modeling of the adsorption process, and metal interactions between the adsorbate and the adsorbent^26^. Pseudo-first-order kinetic (PFO) model, pseudo-second-order kinetic (PSO) model and intraparticle diffusion (IPD) models were applied to determine the adsorption kinetics of Cr(VI) and ARS on the DAC@SC biosorbent surface.

The PFO model is used for reversible reactions in which an equilibrium is established between the liquid and solid phases. The PFO equation is shown in Eq. (12).

12 $${1}/{\text{q}}_{{\text{t}}} \left( {{\text{ads}}} \right) \, = {\text{ k}}_{{1}} /{\text{q}}_{{{\text{e}}({\text{ads}})}} {\text{t }} + { 1}/{\text{q}}_{{{\text{e}}({\text{ads}})}}$$

The PSO kinetic model is applied in chemical adsorption processes that generate covalent forces through the sharing or exchange of electrons between the biosorbent and the adsorbate. The pseudo-second-order equation is shown in Eq. (13)

13 $${\text{t}}/{\text{q}}_{{{\text{t}}({\text{ads}})}} = {1}/{\text{k}}_{{2}} {\text{q}}_{{\text{e(ads)}}}^{{2}} + \, \left( {{1}/{\text{q}}_{{{\text{e}}({\text{ads}})}} } \right){\text{t}}$$

The intraparticle diffusion model (IPD) is shown in Eq. (14):

14 $${q}_{t}={k}_{diff}{\times t}^{0.5}+C$$

where *q*_e_(ads) (mg g^-1^) and *q*_t(ads)_ (mg g^-1^), are the adsorption abilities at equilibrium and at time *t* (min), respectively. K_2_ is the pseudo-second-order adsorption rate constant, *K*_1_ is pseudo-first-order sorption rate constant, and K_diff_ is IPD rate constant. C is the intercept and reflects the boundary layer effect. It was noticed that the contribution of the surface adsorption in the rate-limiting step increased with the intercept increasing.

**Figure 16:**
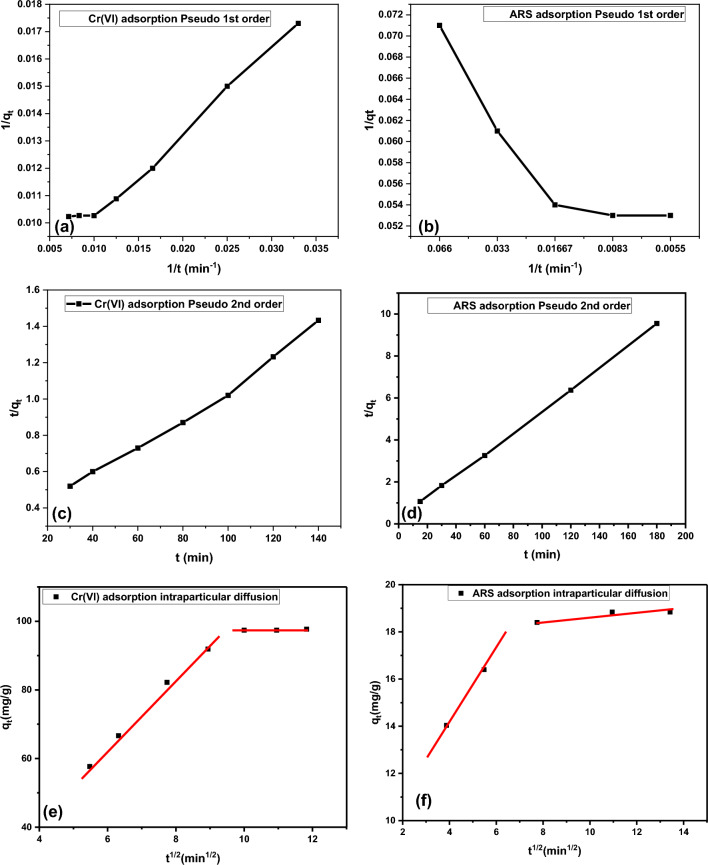
Adsorption kinetics by DAC@SC: (**a**) Pseudo 1st order for Cr(VI), (**b**) Pseudo 1st order for ARS, (**c**) Pseudo 2nd order for Cr(VI), (**d**) Pseudo 2nd order for ARS, (**e**) IPD model for Cr(VI)and (f) IPD model for ARS.

The fit of the experimental data to the PFO, PSO and IPD models is presented in Fig. 16 and the kinetic parameters derived from these models are presented in Table 4. It was observed that the adsorption of Cr(VI) and ARS reached equilibrium within 120 min (2 h) (Fig. 15). When the correlation coefficients (R^2^) of the PFO and PSO models were compared with each other, it was seen that the results fit the PSO kinetic model better. The appearance of two line components instead of a single line passing through the origin in the IPD model graph indicates that adsorption includes different diffusion stages that take place both on the surface and inside the surface. In this case, it was shown that it is not possible to explain the adsorption with a single kinetic model. Figure 16e,f of the IPD model for ARS and Cr(VI) adsorption reveal that the adsorption provides different diffusion stages that occurred on the DAC@SC surface and inside its surface. At first, the adsorption occurred fastly as many active sites are present. Then, with time passing the number of active sites on the DAC@SC biocomposite decreases and the diffusion of Cr(VI) and ARS into pores becomes more difficult so the adsorption becomes slower^64,65^.

**Table 4:** Kinetic parameters for the adsorption of Cr(VI) and ARS onto DAC@SC biosorbent.

Samples	First-order model		
	K_1_ (min^−1^)	q_e1ads_ (mg/g)	R^2^
DAC@SC-Cr(VI)	37.726	130.89	0.983
DAC@SC-ARS	6.9172	20.43	0.986

Samples	Second-order model		
	k_2_ (g/(mg min))	q_e2ads_ (mg/g)	R^2^
DAC@SC-Cr(VI)	2.599 × 10^–4^	123.15	0.991
DAC@SC-ARS	0.01	19.46	0.999

Samples	Intraparticle diffusion model		
	K_diff_	R^2^	
DAC@SC-Cr(VI)	6.55469	0.89895	
DAC@SC-ARS	0.46356	0.75462	

#### Thermodynamic studies

To explore the adsorption process of Cr(VI) and ARS dye onto the DAC@SC biocomposite surface in terms of spontaneity and feasibility and to determine the degree of randomness at the solid/liquid interface, adsorption thermodynamic parameters were determined.

The adsorption of Cr(VI) and ARS was studied at different temperatures from 25 to 45 °C at pH 2 for 2 h. Free energy parameter (ΔG°_ads_), adsorption entropy parameter (ΔS°_ads_), and heat of enthalpy parameter (ΔH°_ads_) of Cr(VI) metal ion, and ARS dye adsorption by DAC@SC adsorbent were calculated. ΔG°_ads_ parameter was calculated from the following equations Eqs. (15), (16) and (17).

15 $${\text{K}}_{{\text{C}}} = {\text{C}}_{{{\text{ad}}}} /{\text{C}}_{{\text{e}}}$$

16 $$\Delta {\text{G}}^{ \circ }_{{{\text{ads}}}} {\text{n }} = \, - {\text{RT ln K}}_{{\text{C}}}$$

17 $$\text{ln K}_\text{C} = \Delta \text{S}^{ \circ}_\text{adsn} /{\text{R}} - \Delta \text{H}^{\circ}_\text{adsn} /\text{RT}$$

where K_c_ is a thermodynamic equilibrium constant, C_ad_ is the adsorbate (Cr(VI) or ARS) concentration taken by DAC@SC material at equilibrium (mg/g), C_e_ is the adsorbate (Cr(VI) or ARS) concentration at equilibrium (mg/L), and R is the universal gas constant.

**Figure 17:**
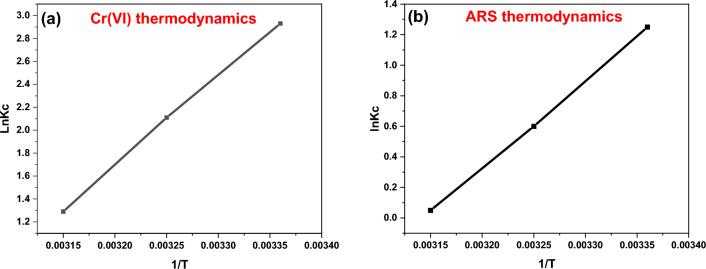
Plot of ln K_C_ vs (1/T) absolute temperature for the adsorption of (**a**) Cr(VI); (**b**) ARS.

The rest of the parameters (ΔH°_adsn_ and ΔS°_adsn_) were calculated from the plot of ln K_c_ vs. 1/T as the slope of the plotting equals (− ΔH°_adsn_/R), and the intercept equals (ΔS°_adsn_/R), Fig. 17.

From the experimental data that present in Table 5, it was noticed that the negative ΔG°_adsn_ value confirms that the adsorption of Cr(VI) and ARS by DAC@SC biocomposite is a spontaneous process. It was also observed that the negative ΔH°_ads_ value confirms that the adsorption of Cr(VI) and ARS by DAC@SC material is exothermic. The negative ΔS°_ads_ values showed that Cr(VI) metal ion, and ARS adsorption onto DAC@SC surface leads to lower disorder and higher arrangement^66–68^.

**Table 5:** Thermodynamic parameters for the biosorption of Cr(VI) and ARS onto DAC@SC.

System	T (k)	K_c_	ΔG°_ads_(KJ/mol)	ΔH°_ads_(KJ/mol)	ΔS°_ads_(J/mol K)
DAC@SC-Cr(VI)	298	18.73	− 7.2593	− 64.879	− 193.53
	308	8.248	− 5.4		
	318	3.633	− 3.41		
DAC@SC-ARS	298	3.48	− 3.0957	− 47.52	− 149.3193
	308	1.822	− 1.536		
	318	1.05	− 0.132		

#### Effect of ionic strength on the adsorption capacity

**Figure 18:**
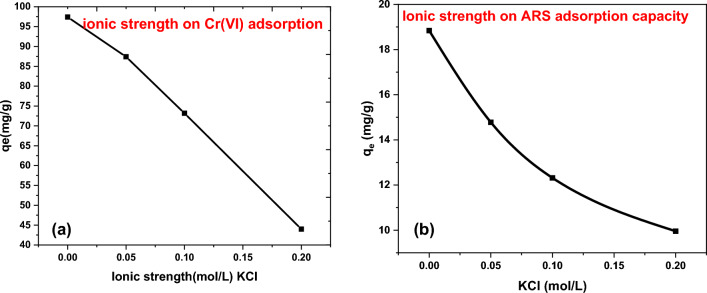
Effect of ionic strength on adsorption of: (**a**) Cr(VI); (**b**) ARS.

Ionic strength parameter was studied by using Cl^−^ inorganic electrolyte in the form KCl. It was investigated by the addition of 0.1 g of DAC@SC respectively to 50 mL aqueous solution of 200 mg/L Cr(VI), 100 mg/L ARS at 25 °C for 3 h and KCl concentration range between 0 and 0.2 mol/L. From experimental data shown in Fig. 18a,b, it can be noticed that with increasing inorganic electrolyte concentration, the adsorption capacity was decreased. A finding which indicated that the presence of inorganic electrolytes suppress the analyte’s adsorption^3^.

#### Desorption and reusability studies

Different eluents were tried for desorption of Cr(VI) and ARS from the DAC@SC as ethanol, HCl (0.2 M), NaOH (0.2 M), NaHCO_3_ (0.1 M) and K_2_CO_3_. It was found that K_2_CO_3_ was the most effective one among them. 0.1 M K_2_CO_3_ was successfully used for desorption of the target adsorbates from DAC@SC biocomposite at room temperature, Table 6.

**Table 6:** Repeated adsorption–desorption cycles for DAC@SC regeneration by using K_2_C_2_O_3_.

Cycle number	Desorption (%)		Recovery (%)	
	Cr(VI)	ARS	Cr(VI)	ARS
1	99.2	99.72	98.9	99.5
2	98.13	95.8	97.3	96.13
3	96.21	95.1	94.8	94.5
4	94	93.27	93.6	92.3
5	91.48	93	92.0	90.08

The re-usability of DAC@SC biocomposite was investigated for five cycles of sorption–desorption sequences under the optimum conditions. From the data presented in Table 6, it can be observed that DAC@SC has high sorption efficiency after five cycles (higher than 90%). So, it is predictable that DAC@SC could be a good sorbent for Cr(VI) and ARS removal from aquatic solutions.

#### Removal of Cr(VI) and ARS from multi-components' solution using DAC@SC biosorbent

**Figure 19:**
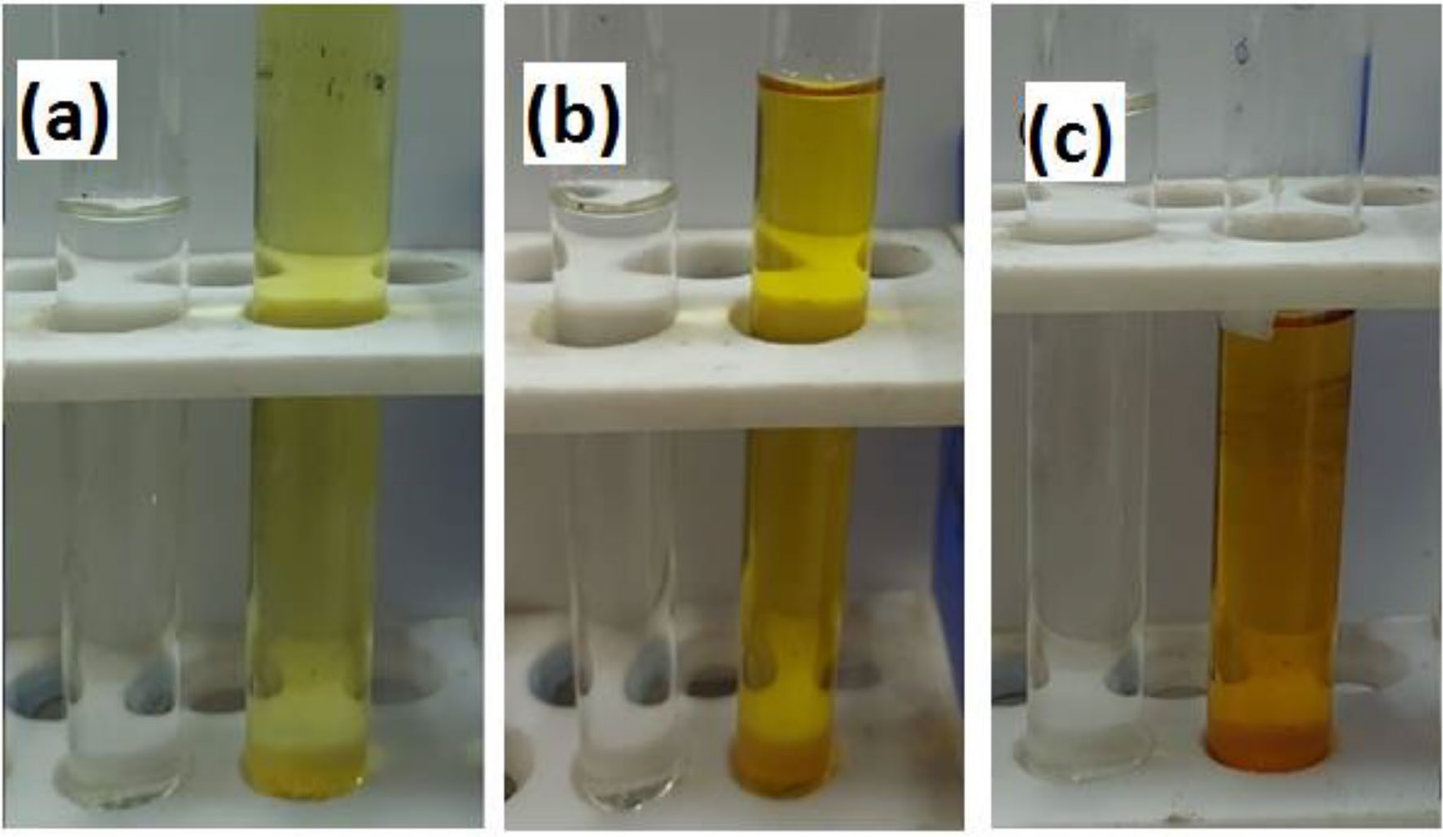
Optical image of (**a**) Cr(VI) solution before and after adsorption, (**b**) ARS solution before and after adsorption, (**c**) mixture of ARS and Cr(VI) solution before and after adsorption.

**Figure 20:**
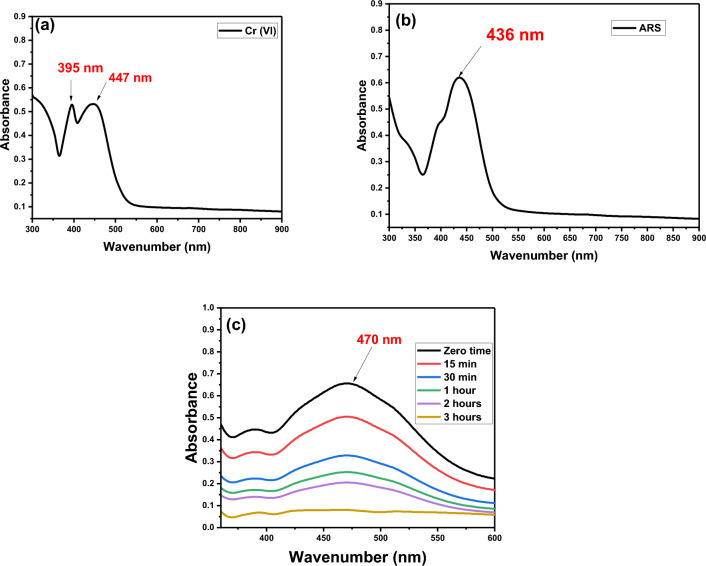
UV spectra of (**a**) of Cr (VI) before adsorption, (**b**) ARS before adsorption (**c**) UV spectra of mixture of Cr (VI) and ARS before and after adsorption by DAC@SC at different time intervals.

As actual wastewater contains different types of organic and inorganic contaminants together, it is important to examine the adsorption capability of synthesized DAC@SC in mixtures. For the adsorption experiment, 0.1 g of DAC@SC was added to 25 mL of each adsorbate solutions (100 ppm) at pH 2. Then the mixture was shaken at 120 rpm for 3 h. The equilibrium concentration of the adsorbates was calculated from UV–Vis data. The adsorption efficiency of anionic adsorbates is calculated using Eq. (2). The optical images of Cr(VI), ARS, Cr(VI) + ARS solutions before and after adsorption in single and binary manner are shown in Fig. 19a–c. Obvious color changes can be noticed before and after adsorption of the single anion and mixed anion solutions. The absorption spectra of Cr(VI) and ARS solutions before adsorption and the absorption spectrum of the remain after adsorption are presented in Fig. 20a–c. For Cr(VI) the UV–Vis spectrum(Fig. 20a) shows two peaks at 395 and 447 nm while the UV–Vis spectrum of ARS(Fig. 20b) shows maximum peak at 436 nm. On the other hand, Fig. 20c shows that when the two anionic pollutants were mixed, overlapping of the peaks occurred and a new peak appeared at 470 nm. It was observed that at time 3 h, the new peak was completely disappeared. Additionally, the concentration of each of Cr(VI) and ARS in the remaining after adsorption of the mixture was determined. More than 95% of both Cr(VI) and ARS were simultaneously removed by the DAC@SC biocomposite.

#### Plausible mechanism of biosorption of Cr(VI) and ARS onto DAC@SC biosorbent

**Figure 21:**
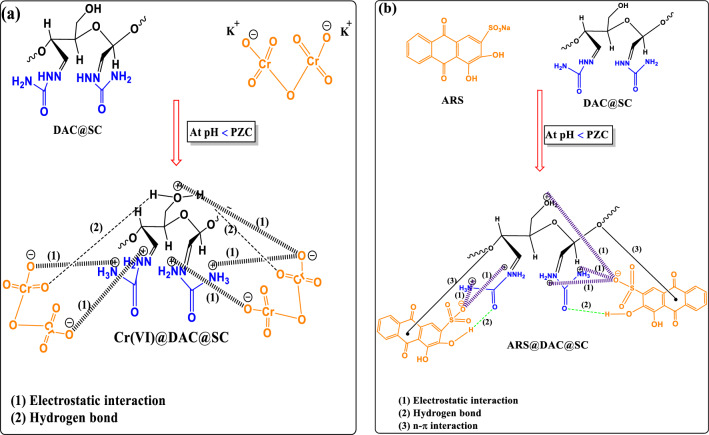
Plausible mechanism of biosorption of (**a**) Cr(VI) ions and (**b**) ARS onto DAC@SC.

To investigate the possible mechanism of Cr(VI) adsorption on DAC@SC, morphology, surface charge and FTIR of the adsorbents were evaluated. The adsorption mechanism of the Cr(VI) and ARS anionic dye was designed in light of the effective groups available on DAC@SC surface as shown in Fig. 21. In fact, DAC@SC biocomposite is very abundant with active groups that can adsorb both anionic pollutants. These active groups come from the fact that the adsorbent is composed of dialdehyde cellulose and semicarbazide which in their origin are rich in active groups. Thus, the active groups are amino (–NH_2_), carbonyl, hydroxyl (-OH). In the acidic medium, the DAC@SC biocomposite acts on the adsorption of the Cr(VI) through the electrostatic interactions between the negatively charged oxygen on Cr(VI) and the positively charged groups (–NH_3_^+^, –OH^+^) of the DAC@SC biocomposite, Fig. 21a.

On the other hand, in the acidic medium, the DAC@SC biocomposite acts on the adsorption of the ARS dye through the electrostatic interactions between the sulfonate groups (–SO_3_^−^) of the ARS dye and the positively charged groups (–NH_3_^+^, –OH^+^) of the DAC@SC biocomposite, Fig. 21b. In addition, hydrogen interactions also play a vital role in the adsorption process for both species through the interaction between hydrogen on the surface of DAC@SC adsorbent and atoms including oxygen and nitrogen in the structure of the Cr(VI) and ARS, respectively^69^.

Finally, n–π’s interactions contribute to the adsorption process of ARS dye through the interaction between the electron-donating system represented by the nitrogen and oxygen groups in DAC@SC adsorbent and the electron-gaining system represented by the aromatic rings of ARS dye^70^, Fig. 21b.

#### Applications

##### Analysis of wastewater samples

Analysis of wastewater samples was used for offered method accuracy confirmation. From the experimental results present in Table 7, it was noted that a good covenant was achieved between the added adsorbate concentration and the obtained one by using the test procedure. The recoveries higher than 90% show that the anticipated procedure gives a suitable accuracy on real samples analysis.

**Table 7:** Analysis of Cr(VI) and ARS in spiked wastewater samples by DAC@SC biosorbent (n = 3).

Wastewater samples locations	Species	Added (µg/mL)	Found (µg/mL)	Recovery (%)	RSD (%)
Mansoura	Cr(VI)	00.0	00.0	00.0	00.0
		100.0	4.60	97.34	1.19
		150	17.04	90.08	1.22
Sinbellawin		00.0	0.00	0.00	0.00
		100.0	3.98	97.97	1.27
		150	12.71	93.01	1.30
Mokataa		0.00	0.00	0.00	0.00
		100.0	4.1	97.85	1.25
		150	10.935	94.22	1.23
Talkha		0.00	0.00	0.00	0.00
		100	5.43	96.68	1.26
		150	9.45	95.23	1.24
Manzalla		0.00	0.00	0.00	0.00
		100.0	3.1	98.87	1.27
		150	7.83	96.32	1.29
Mansoura	ARS	0.00	0.00	0.00	0.00
		50.0	2.34	96.89	1.17
		100	6.8	98.9	1.19
Sinbellawin		0.00	0.00	0.00	0.00
		50.0	4.28	92.94	1.25
		100	12.03	93.38	1.23
Mokataa		0.00	0.00	0.00	0.00
		50.0	4.18	91.64	1.23
		100	9.27	96.316	1.26
Talkha		0.00	0.00	0.00	0.00
		50.0	4.62	92.25	1.3
		100	12.53	92.85	1.2
Manzalla		0.00	0.00	0.00	0.00
		50.0	3.55	94.42	1.27
		100	11.985	93.43	1.25

##### Removal  of Cr(VI) and ARS from simulated synthetic effluents

The prepared DAC@SC biosrbent was tested for decontamination of synthetic effluents to check the usefulness of DAC@SC biosorbent in real applications. Two simulated synthetic effluents were prepared as defined in Table 8. The synthetic effluents are consisting of various surfactants and salts. Each sample was spiked with different amounts of Cr(VI) and ARS and the process of SPE and determination of Cr(VI) and ARS was performed as previously mentioned. The results obtain are shown in Table 8. As it can be noticed, the DAC@SC was able to remove more than 95% of Cr(VI) and ARS from effluent A and B. Based on these results, we can conclude that the DAC@SC could be used successfully in the field of real wastewater treatment.

**Table 8:** Determination of Cr(VI) and ARS in synthetic effluents by biosorption using 5 mg DAC@SC biocomposite at pH 2.0 (n = 5).

Synthetic effluents (µg/mL)	Species	Determined	Spiked (µg/mL)	Measured (µg/mL)	R, (%)	RSD (%)
Effluent A NaCl(10), KCl (10), Na_2_SO_4_(10), KNO_3_(10), Na_2_CO_3_(10), Sodium Dodecyl Sulfate (10), CH_3_COONa (10), (NH_4_)_2_PO_4_ (10)	Cr(VI)	10.0	5.00	14.80	96.00	2.07
			10.0	19.70	97.00	1.19
			15.0	24.80	98.67	1.22
Effluent B NaCl(20), KCl (20), Na_2_SO_4_(20), KNO_3_(20), Na_2_CO_3_(20), Sodium dodecyl Sulfate (20), CH_3_COONa (20), (NH_4_)_2_PO_4_ (20)		20.0	5.00	24.90	98.00	1.95
			10.0	29.75	97.50	1.27
			15.0	34.78	98.50	1.30
Effluent A NaCl(10), KCl (10), Na_2_SO_4_(10), KNO_3_(10), Na_2_CO_3_(10), Sodium dodecyl Sulfate (10), CH_3_COONa (10), (NH_4_)_2_PO_4_ (10)	ARS	20.0	5.00	24.85	97.00	2.30
			10.0	29.34	93.40	1.17
			15.0	34.80	98.67	1.19
Effluent B NaCl(20), KCl (20), Na_2_SO_4_(20), KNO_3_(20), Na_2_CO_3_(20), Sodium dodecyl Sulfate (20), CH_3_COONa (20), (NH_4_)_2_PO_4_ (20)		40.0	5.00	44.8	96.00	2.04
			10.0	49.70	97.00	1.25
			15.0	54.8	98.66	1.23

#### Performance of the prepared DAC@SC

In order to enhance the value of our adsorbent, we carried out a comparative study of the maximum adsorption capacity obtained for the same pollutant to other adsorbents and activated carbon reported in the literature. Table 9 grouped together the different values of q_max_ for the different adsorbents. We can see that the Cr(VI) and ARS adsorption observed in the present study is well positioned with respect to other researches with a maximum adsorption capacity q_max_ of 97.4 for Cr(VI) and 18.84 for ARS at 298 K, relatively, interesting compared to other adsorbents. The differences of the Cr(VI) and ARS uptakes are due to the morphological properties of each adsorbent like the structure, the functional groups and the surface area. DAC@SC could be an attractive adsorbent for anionic species owing to its isoelectric point pHpzc. Desorption is an unavoidable process and is an intermediate stage toward the adsorbent regeneration. The latter is an essential point to estimate the reutilization of any adsorbent for industrial applications, owing to the ecological concerns and the needs for sustainable development. In the future, column scale and pilot plant experiments can be implemented to be applied in the wastewater treatment plant for cationic and anionic metal ions and textile dye removal from wastewater.

**Table 9:** Comparison of equilibrium time of various adsorbents for Cr(VI) and ARS.

Anion species	Adsorbent	Adsorbent dose	Initial concentration (ppm)	Equilibrium time	Sorption capacity (mg/g)	Ref.
Cr(VI)	Pineapple peel derived biochars	5 g/L	10	8 h	41.67	^71^
	sugarcane bagasse magnetic biochar	0.1 g/25 mL	100	24 h	43.122	^72^
	Fe3O4 @ Cr(VI) IIPs	80 mg/10 mL	12	30 min	2.49	^73^
	Fe3O4 @ Cr(VI) NIPs	80 mg/10 mL	12	30 min	0.52	^74^
	Cellulose-1N	6.1 mg/20 mL	50	300 min	10.2	^75^
	Cellulose-2N	6.1 mg/20 mL	50	300 min	18.1	^75^
	Cellulose-3N	6.1 mg/20 mL	50	300 min	34.7	^75^
	DAC@SC	0.1 g/50 mL	200	100 min	97.4	Present study
ARS	APTES grafted sonicated vermiculite	0.8 mg/mL	0.016mmolL − 1	40 min	18.2	^76^
	*S. platensis*	1.5 g/L	100 ppm	42.5 min	17.15	^77^
	Chitosan/ZnO nan°Composite	0.1 g/50 mL	20 ppm	48 h	8.01	^78^
	Olive stone	5 g/L	110 mg/L	72 h	16.1	^79^
	Lantana camara	0.5 g/50 mL	25 ppm	90 min	1.165	^80^
	Mustard husk	0.5 g/50 mL	25 ppm	80 min	1.97	^81^
	DAC@SC biocomposite	0.15/50 mL	100 ppm	100 min	18.84	Present study

## Conclusion

In this work, flax fiber based semicarbazide biosorbent was prepared in two successive steps. In the first step, flax fibers were oxidized using potassium periodate (KIO_4_) to yield diadehyde cellulose (DAC). Dialdehyde cellulose was, then, refluxed with semicarbazide.HCl to produce the semicarbazide functionalized dialdehyde cellulose (DAC@SC). The prepared DAC@SC biocomposite was characterized by the BET, elemental analysis, FTIR, ^1^HNMR, SEM, TEM, TGA and XRD methods. The efficiency of the DAC@SC biocomposite was studied for the removal of Cr(VI) and ARS from aqueous solutions; the influence of the initial pH, Cr(VI) and ARS concentration, contact time, adsorbent dose and temperature on adsorption of Cr(VI) and ARS were investigated. When pH is 2, oscillation time is 120 min, and temperature is 25 °C, the maximum adsorption capacity was found to be 97.4 mg/g and 18.84 mg/g for Cr(VI) and ARS, respectively. The adsorption process here was well matched to PSO and Langmuir models. The 8 < E_DR_ > 16 kJ/mol determined according to the D–R isotherm showed that the biosorption proceeded chemically. The negative free enthalpy ΔG° and negative enthalpy ΔH° indicated that the adsorption of Cr(VI) and ARS onto DAC@SC is spontaneous and exothermic over the studied temperatures range. The prepared DAC@SC was regenerated using K_2_CO_3_ eluent. The plausible mechanism of adsorption of Cr(VI) and ARS dye on the surface of DAC@SC biocomposite is supposed to proceed through chemical interactions, the most prominent of which are electrostatic, H-bonding, and n–π interactions. This work points out that DAC@SC biocomposite could be used as a promising and effective adsorbent biosorbent for the removal of toxic anionic species from wastewater.

The comparison of the maximum biosorption capacity of DAC@SC biosorbent with other sorbents used in Cr(VI) and ARS removal reported in the literature is given in Table 9. According to these results, it was determined that DAC@SC biosorbent is an effective biosorbent for Cr(VI) and ARS removal. In addition, due to the fact that flax is abundant, low cost and renewable due to being agricultural waste, DAC@SC biosorbent can be used as an alternative biosorbent in the removal of dyes and metal ions in wastewater.

**Figure 22:**
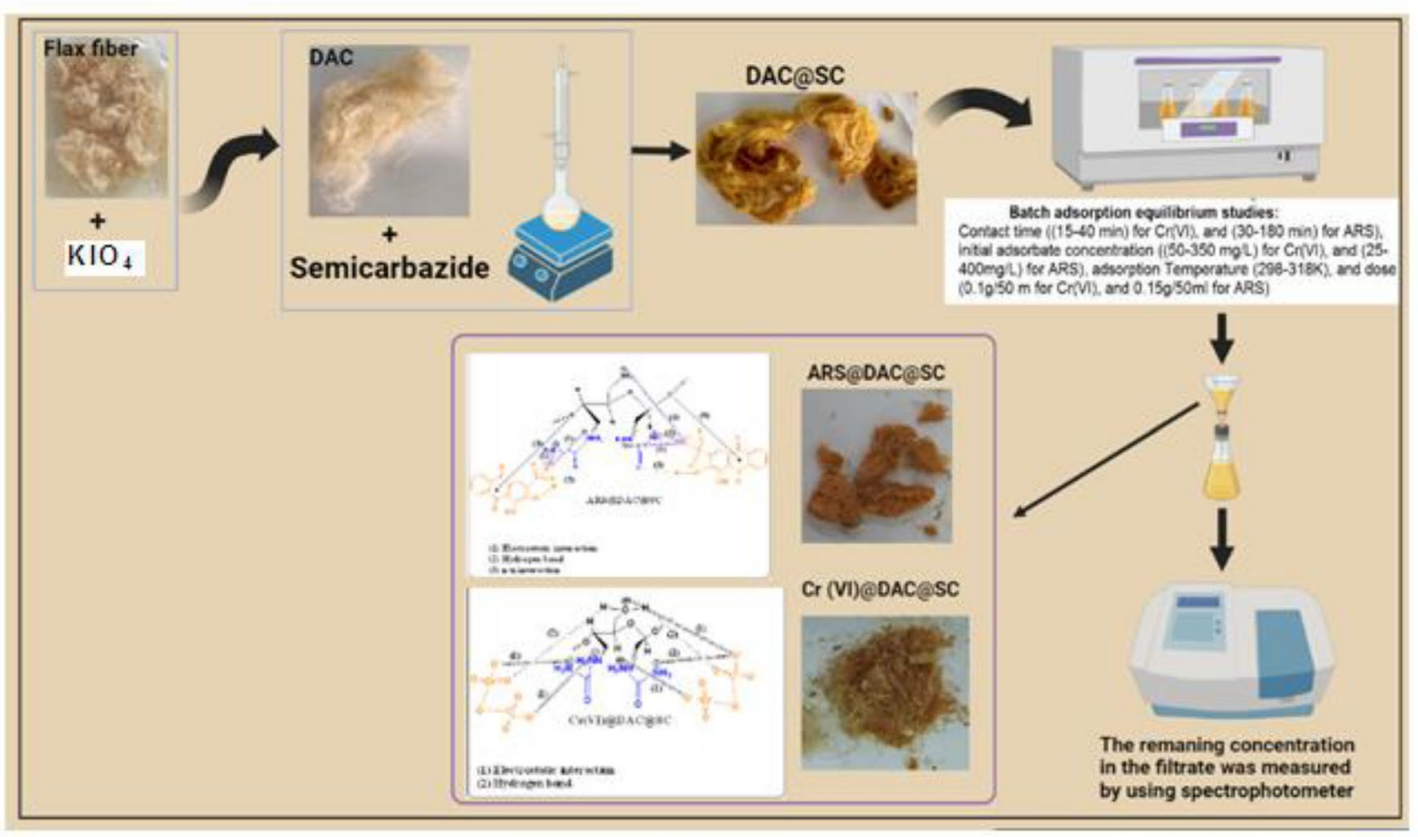
Synthesis of DAC@SC and its use for biosorption of Cr(VI) and ARS.

Finally, the process of synthesis of DAC@SC biosorbent, biosorption of analytes and the plausible mechanism of biosorption are represented in Fig. 22.

## Data Availability

All data generated or analysed during this study are included in this published article.
